# Adjunctive cytoprotective therapies in acute ischemic stroke: a systematic review

**DOI:** 10.1186/s12987-021-00280-1

**Published:** 2021-10-19

**Authors:** I. A. Mulder, E. T. van Bavel, H. E. de Vries, J. M. Coutinho

**Affiliations:** 1 Department of Biomedical Engineering and Physics, Amsterdam Cardiovascular Sciences, Amsterdam UMC, University of Amsterdam, Amsterdam, The Netherlands; 2 Department of Molecular Cell Biology and Immunology, Amsterdam Neuroscience, Amsterdam UMC, Vrije Universiteit Amsterdam, Amsterdam, The Netherlands; 3 Department of Neurology, Amsterdam Neuroscience, Amsterdam UMC, University of Amsterdam, Amsterdam, The Netherlands

## Abstract

With the introduction of endovascular thrombectomy (EVT), a new era for treatment of acute ischemic stroke (AIS) has arrived. However, despite the much larger recanalization rate as compared to thrombolysis alone, final outcome remains far from ideal. This raises the question if some of the previously tested neuroprotective drugs warrant re-evaluation, since these compounds were all tested in studies where large-vessel recanalization was rarely achieved in the acute phase. This review provides an overview of compounds tested in clinical AIS trials and gives insight into which of these drugs warrant a re-evaluation as an add-on therapy for AIS in the era of EVT. A literature search was performed using the search terms “ischemic stroke brain” in title/abstract, and additional filters. After exclusion of papers using pre-defined selection criteria, a total of 89 trials were eligible for review which reported on 56 unique compounds. Trial compounds were divided into 6 categories based on their perceived mode of action: systemic haemodynamics, excitotoxicity, neuro-inflammation, blood–brain barrier and vasogenic edema, oxidative and nitrosative stress, neurogenesis/-regeneration and -recovery. Main trial outcomes and safety issues are summarized and promising compounds for re-evaluation are highlighted. Looking at group effect, drugs intervening with oxidative and nitrosative stress and neurogenesis/-regeneration and -recovery appear to have a favourable safety profile and show the most promising results regarding efficacy. Finally, possible theories behind individual and group effects are discussed and recommendation for promising treatment strategies are described.

## Significance statement

Dozens of clinical stroke trials have been performed in the search for additional therapeutic strategies next to thrombolysis, but all failed to consistently improve outcome. With the introduction of endovascular thrombectomy, a new era for treatment of AIS has arrived. We summarized therapeutic strategies and clinical trial results. This review will function as an important enchiridion for future clinical stroke trials and it will provide an insight into which of these drugs warrant a re-evaluation in combination with thrombectomy.

## Introduction

About 25 years ago the NINDS trial established intravenous thrombolysis (IVT) with tPA as the first effective medical therapy for acute ischemic stroke (AIS) [1]. Still, a large proportion of patients are not eligible for, or do not benefit from IVT. In the following years, dozens of clinical trials have been performed in the search for additional therapeutic strategies to reduce infarct volume and improve clinical outcome. Preclinical evidence is usually the trigger for making the clinical translation. Unfortunately, none of the compounds tested in these trials consistently showed to improve patient outcome, despite promising pre-clinical data, illustrating a translational gap [2]

With the introduction of endovascular thrombectomy (EVT), a new era for treatment of AIS has arrived [3]. With EVT, rapid recanalization of the major vessels can be achieved in the vast majority of the patients with a large vessel occlusion [4]. Up to 38% of all acute ischemic strokes are large vessel occlusions [5]. Despite the much larger recanalization rate as compared to IVT alone, final outcome remains far from ideal, with approximately 50% of patients having a poor outcome at 90 days [4]. This raises the question if some of the previously tested neuroprotective drugs warrant re-evaluation, since these compounds were all tested in studies where large-vessel recanalization was rarely achieved in the acute phase. This question was also discussed in two extensive reviews by Savitz et al*.* [6, 7], where the whole aspect of “if, how and when” additional therapies next to reperfusion could be useful, is elaborated including recommendations and guidelines for clinical and per-clinical stroke trials.

After large vessel occlusion in the brain occurs, several multi-phased cascades start to unroll, with necrosis as devastating endpoints. Although these cascades are all intertwined and interact with one another, several main mechanisms can be identified, namely compounds acting on systemic haemodynamic, excitotoxicity, oxidative (and nitrosative) stress, neuro-inflammation and blood–brain barrier damage and vasogenic edema. In this review, we discuss the therapeutic strategies to target these pathways, and systematically review the results from clinical trials in which these various compounds have been tested. This review aims to provide an insight into which of these drugs may warrant a re-evaluation as an add-on therapy for acute ischemic stroke in the era of EVT.

## Methods

All aspects of the review methodology, analysis and reporting were carried out based on the AMSTAR [8] and PRISMA [9] guidelines, with the exception that screening and selection was done by a single author.

### Literature search strategy

A literature search was performed in Pubmed using the search terms “ISCHEMIC STROKE BRAIN” in title/abstract, and the following Pubmed filter options were activated: “Clinical Trial”, “Randomized Controlled Trial” and “Humans”. The search was limited to full length papers written in English. The initial Pubmed search was conducted between March 2018–May 2019 and we selected studies published until 2019. Potentially relevant papers were selected using title and abstract screening, after which full text evaluation was performed. A list of included papers and their described treatment strategies was compiled from the literature search, after which all selected compounds were additionally crosschecked with ClinicalTrials.gov and other reviews for possible additional trials missed by the initial search. Clinical trial numbers were then again checked in Pubmed to make sure that the initial search included all relevant papers. Only clinical trials with published results were included in this review.

### Study selection

Clinical trials were excluded if no additional therapy was tested besides reperfusion therapy (IVT/EVT) or antiplatelet therapy. Studies with therapies other than clearly defined chemicals (e.g. hypothermia, high-pressure oxygen, haemodilution or herbs-mixtures) were also excluded. In addition, stroke prevention trials and treatment in the chronic phase (defined as start treatment after 4 days post stroke onset) were excluded. As a quality control, only randomized (placebo) controlled trials with > 15 patients per study group were included. In order to be eligible, trials had to have reported at least one of the following outcome scores: modified Rankin Scale (mRS), National Institutes of Health Stroke Scale (NIHSS), Barthel Index (BI), Glasgow Outcome Score (GOS), (modified) Mathew scale (all scoring details available at http://www.strokecenter.org) [10, 11] or the radiological outcome measure infarct volume or growth on CT or MRI.

Trial compounds were divided into 6 categories based on their perceived mode of action: systemic haemodynamics, excitotoxicity, neuro-inflammation, blood–brain barrier and vasogenic edema, oxidative and nitrosative stress, neurogenesis, and regeneration. A 7th category was composed of compounds that did not fit in one of the other categories. Compounds with overlapping functionality were categorized according to their presumed primary working mechanism.

### Data collection

Relevant data were extracted from each study, using only the main publication of each study. A complete list of subgroup analyses and other post-hoc analyses is beyond the scope of this review. As main clinical outcome, the mRS score (and readout time point) is stated. If mRS was not provided, we used the NIHSS, BI, mortality or infarct volume (in descending order). The effect size (in mean, median, percentage or ratio) is stated for placebo vs study drug, with accompanying (unadjusted) statistical certainty (p-value or 95% confidence interval). Additionally, the presence of safety concerns is stated (yes or no), based on any significant difference described for each study concerning (serious) adverse events or mortality.

## Results

**Fig. 1:**
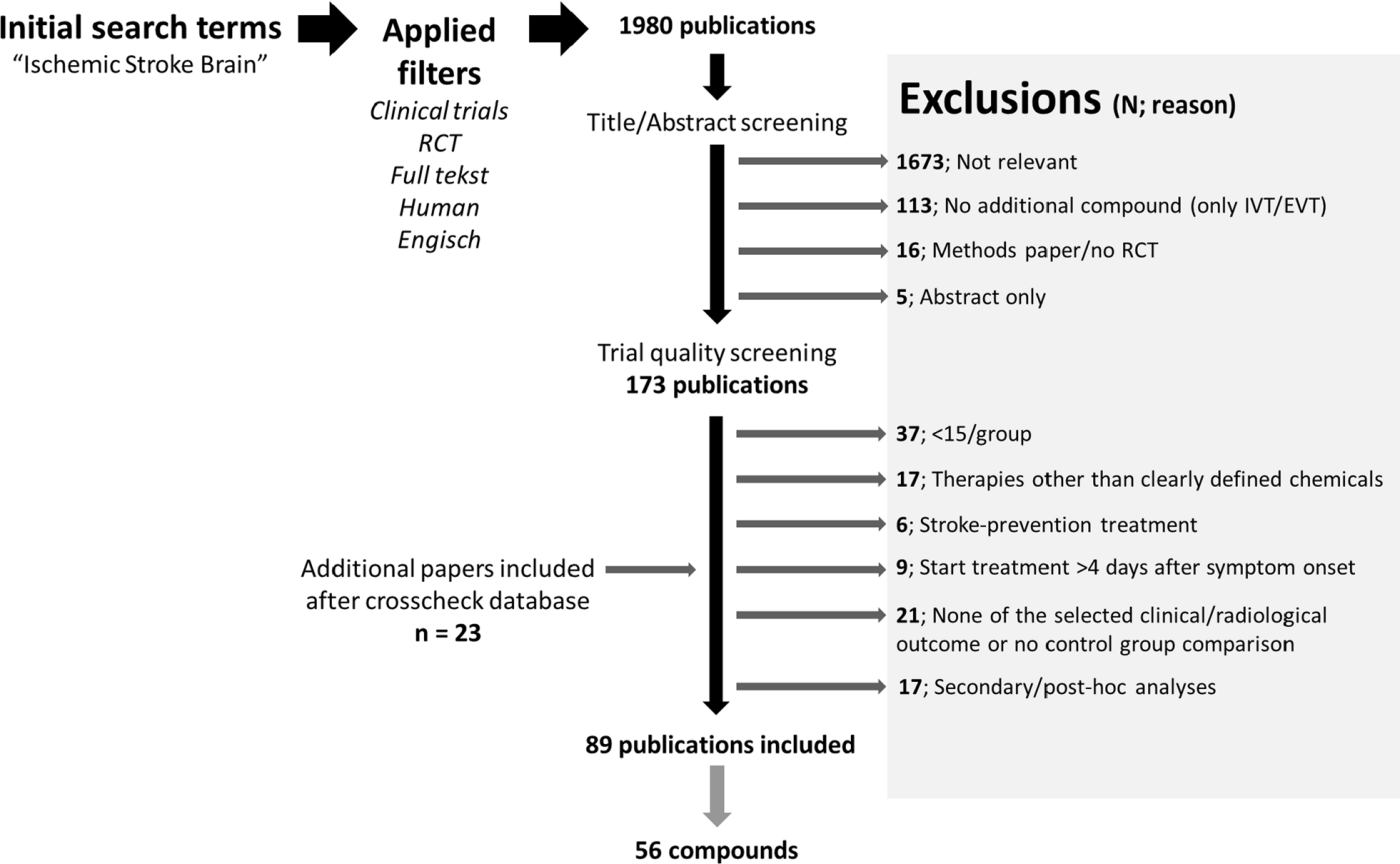
Flowchart of the in- and excluded compounds tested in clinical stroke trials. *RCT *randomized controlled trial, *N* number of papers, *IVT* intravenous thrombolysis, *EVT *endovascular thrombectomy

The initial Pubmed search resulted in a total of 1980 papers, of which 173 were selected for further review. After exclusion of papers using the listed criteria, and after crosscheck with ClinicalTrials.gov, a total of 89 articles were eligible for review which reported on 56 unique compounds (Fig. 1). Compounds are discussed per category, in alphabetic order. A list of included studies can be found in Table 1.

**Table 1:** Overview of included randomized clinical trials per compound and per category. Trial compounds showing a positive treatment effect over control groups are highlighted (bold)

Compound	Mechanism of action	Year	Blinding$^{\infty }$	N$^{\omega }$	IVT/EVT$^{\dagger }$	Age$^{\lambda }$	NIHSS$^{\rho }$	T-t-T$^{\xi }$	Main outcome parameter	Effect size$^{\pi }$	P or ratio [CI]$^{\ddagger }$	Safety concern
						(y)		(h)	(time point)			
**Systemic heamodynamics**												
Atenolo [13]	${\beta }_{1}$-blocking agent	1988	++	201	+/−	69	?	24	Mortality (30 d)	14 ${\text{vs}}$ 25	?	Yes
Candesartan [14]	AngII-R antagonist	2003	++	339	?	68	?	30	BI (30 d)	89 ${\text{vs}}$ 87	ns	No
Candesartan [16]		2011	++	2029	+/−	71	?	18	mRS (6 mo)	2.0 ${\text{vs}}$ 2.1	1.13 (OR) [0.97–1.32]$^{a}$	No
DCLHb [18]	Increase blood oxygen level	1999	+	85	+/−	66	?	?	mRS $\ge$3 (90 d)	51% ${\text{vs}}$ 85%	0.002	Yes
Irbesartan [19]	AngII-R antagonist	2011	++	43	+/−	69	7	53	Infarct volume (30 d)	280 ${\text{vs}}$ 822	0.63$^{a}$	No
Lifarizine [20]	Sodium and calcium antagonist	1996	++	117	?	69	?	?	Independent mRS (90 d)	$\Delta$placebo-drug = 16%	0.522	No
Lisinopri [21]	ACE inhibitor	2007	++	40	?	74	11	19	mRS $\le$2 (90 d)	63% ${\text{vs}}$ 60%	0.56$^{a}$	No
Magnesium sulfate [22]	Multiple action sides resulting in vasodilatation and increased CBF	2004	++	2386	+/−	70	?	7	mRS $\ge$2 (90 d)	72% ${\text{vs}}$ 70%	0.95 (OR) [0.80–1.13]	Yes
Magnesium sulfate [23]		2015	++	1700	+/−	69	11	45 min	mRS (90 d)	2.7 ${\text{vs}}$ 2.7	1$^{a}$	No
** Nimodipine** [24]	Calcium antagonist	**1988**	**++**	**186**	**−**	**69**	**?**	**?**	**Mortality (6 mo)**	**28**% ${\text{vs}}$ **17%**	**0.046**	**No**
** Nimodipine** [24]		**1989**	**++**	**41**	**−**	**63**	**?**	**?**	**mMS (28 d)**	**78 **${\text{vs}}$ **89**	<**0.04**	**No**
Nimodipine [195]		1990	++	52	?	65	?	19	MS improvement (4 mo)	86% ${\text{vs}}$ 75%	ns	No
Nimodipine [196]		1990	++	1215	?	73	?	?	BI $\ge$60 (6 mo)	58% ${\text{vs}}$ 55%	0.31	No
Nimodipine [197]		1992	++	1064	?	66	?	28	Mortality (6 mo)	16% ${\text{vs}}$ 14%	ns	No
Nimodipine [198]		1993	++	164	?	72	?	?	Mortality (28 d)	15% ${\text{vs}}$ 10%	ns	No
Nimodipine [27]		1994	++	350	?	58	?	20	mRS $\le$2 (12 mo)	64% ${\text{vs}}$ 66%	ns	Yes
** Nimodipine** [26]		**1994**	**++**	**295**	**?**	**72**	**?**	**11**	**BI (21 d)**	**22** ${\text{vs}}$ **10**	**0.003**$^{a}$	**No**
Nimodipine [199]		2000	++	153	?	59	?	?	Infarct volume (90 d)	10.2 ${\text{vs}}$ 4.4	ns	?
Nimodipine [200]		2001	++	454	?	71	?	?	mRS >3 (90 d)	27% ${\text{vs}}$ 32%	1.2 (RR) [0.9−1.6]	No
Piracetam [28]	Positive allosteric AMPA-R modulator	1997	++	927	−	70	?	7	BI (90 d)	53 ${\text{vs}}$ 56	0.33	No
Propranolol [13]	$\beta$-blocking agent	1988	++	201	+/−	68	?	25	Mortality (30 d)	14 ${\text{vs}}$ 27	?	Yes
**Excitotoxicity**												
Aptiganel HCl [34]	NMDA-R antagonist	2001	++	628	−	72	12	?	Phase II part: $\Delta$NIHSS (7 d)	− 4 ${\text{vs}}$− 3.5	0.41	Yes
									Phase III part: mRS (90 d)	3.1 ${\text{vs}}$ 3.3	0.44	
Clomethiazole [35]	GABA-R agonist	1999	++	1360	−	71	?	?	BI $\ge$60 (90 d)	55% ${\text{vs}}$ 56%	0.649	No
Clomethiazole [37]		2001	++	190	+	67	14	5.4	BI $\ge$60 (90 d)	67% ${\text{vs}}$ 66%	0.94 (OR) [0.51–1.72]	No
Clomethiazole [36]		2002	++	1198	−	72	17	?	BI $\ge$60 (90 d)	46% ${\text{vs}}$ 42%	0.11	No
Diazepam [38]	GABA-R agonist	2006	++	849	?	72	?	?	mRS <3 (90 d)	49% ${\text{vs}}$ 52%	1.14 (OR) [0.87−1.49]	No
Fanapanel [39]	AMPA-R antagonist	2002	++	61	–	67	6	17	NIHSS (30 d)	?	0.07	Yes
Gavestinel (GV150526) [40]	NMDA-R antagonist	2001	++	1367	?	72	12	5.2	mRS $\le$1 (90 d)	26% ${\text{vs}}$ 29%	0.3	No
Lanicemine HCl [41]	NMDA-R antagonist	2002	++	103	–	67	9	?	$\Delta$NIHSS $\ge$4 (30 d)	?	0.563 (OR) [0.24−1.31]	Yes
Lithium [42]	NMDA-R antagonist	2014	++	66	+/–	62	7	?	$\Delta$NIHSS (30 d)	− 1.64 ${\text{vs}}$ − 2.34	0.402	No
Lubeluzole [45]	Indirect NMDA-R antagonist	1996	++	193	?	71	15	4.3	$\Delta$NIHSS (28 d)	− 5.8 ${\text{vs}}$ − 6.4	ns	Yes
** Lubeluzole** [43]		**1997**	**++**	**700**	**+/−**	**71**	**15**	**4.6**	**mRS** $\ge$**3 (90 d)**	**44%** ${\text{vs}}$ **42%**	**0.04**	**No**
Lubeluzole [44]		1998	++	725	−	70	?	4.4	mRS 3−5 (90 d)	48% ${\text{vs}}$ 52%	ns	No
Nalmefene [46]	Opioid-R antagonist	2000	++	330	+/–	70	12	4.4	$\Delta$NIHSS $\ge$4 (90 d)	65% ${\text{vs}}$ 64%	0.88	No
Selfotel [48]	NMDA-R antagonist	2000	++	567	?	69	14	4.5	BI $\ge$60 (90 d)	61% ${\text{vs}}$ 58%	0.49	Yes
**Oxidative and nitrosative stress**												
Disufenton sodium [50]	Free radical scavenger	2001	++	147	−	68	8	15	$\Delta$NIHSS (30 d)	− 3 ${\text{vs}}$ − 3	ns	No
** Disufenton sodium** [53]		**2006**	**++**	**1699**	**+/−**	**68**	**13**	**3.8**	**mRS (90 d)**	$\le$**1: 31%** ${\text{vs}}$ **33%**	**1.20 (OR) [1.01−1.42]**	**No**
Disufenton sodium [54]		2007	++	3195	+/–	69	13	3.8	mRS (90 d)	$\le$1: 29% ${\text{vs}}$ 27%	0.94 (OR) [0.83−1.06]$^{a}$	No
Disufenton sodium [51]		2008	++	5028	+/−	71	13	3.8	mRS (90 d)	$\le$1: 29% ${\text{vs}}$ 29%	1.02 (OR) [0.92−1.13]$^{a}$	No
** Ebselen** [58]	Free radical scavenger	**1998**	**++**	**300**	**−**	**65**	**?**	**29**	**BI** $\ge$**75 (90 d)**	**50%** ${\text{vs}}$ **64%**	**0.012**	**No**
** Ebselen** [57]		**1999**	**++**	**99**	**+/−**	**99**	**?**	**9**	**Infarct volume (30 d)**	**12.74** ${\varvec{{\text{vs}}}}$ **8.30**	**0.034**	**No**
** Edaravone** [59]	Free radical scavenger	**2003**	**++**	**250**	**−**	**66**	**7**	**36**	**mRS (90 d)**	**2.2** ${\varvec{{\text{vs}}}}$ **1.8**	**0.038**	**No**
Epigallocatechin Gallate [60]	Free radical scavenger	2017	++	71	+	65	15	?	NIHSS $\le$10 (7 d)	41% ${\text{vs}}$ 45%	ns	No
Glyceryl Trinitrate [61]	Free radical scavenger	2015	++	4011	+/−	70	11	26	mRS (90 d)	?	1.01 (OR) [0.91–1.13]$^{a}$	No
Glyceryl Trinitrate [63]		2019	+	852	+/−	74	10	1.1	mRS (90 d)	3 ${\text{vs}}$ 3	0.083$^{a}$	No
** Uric−acid** [68]	Free radical scavenger	**2014**	**++**	**411**	**+**	**77**	**13**	**3**	**mRS (90 d)**	**3** ${\varvec{{\text{vs}}}}$ **2**	**0.045**$^{a}$	**No**
Tirilazad mesylate [70]	Free radical scavenger	1994	++	111	+/−	66	8	8.5	NIHSS (90 d)	1 ${\text{vs}}$ 2	0.105	No
Tirilazad mesylate [71]		1996	++	556	−	69	10	4.3	BI (90 d)	95 ${\text{vs}}$ 95	0.87 (OR) [0.60−1.25]$^{a}$	No
Tirilazad mesylate [72]		2002	+	368	?	70	?	?	Infarct volume (8 d)	37mL ${\text{vs}}$ 43mL	0.55	?
**Blood–brain barrier and vasogenic edema**												
Albumin+Ringers-glucose [10]	Colloid-osmotic pressure modifier	1992	−	297	+/−	75	?	?	Mortality (90 d)	21% ${\text{vs}}$ 17%	ns	No
Albumin [80]		2011	++	424	+/−	70	11	3.5	Mortality (90 d)	13% ${\text{vs}}$ 21%	1.55 (RR) [1.01−2.39]	Yes
Albumin [77]		2013	++	841	+/−	64	11	?	Good outcome (90 d)	44% ${\text{vs}}$ 44%	0.96 (RR) [0.84−1.10]$^{a}$	Yes
** Citicoline** [83]	Cell membrane stabilizer	**1997**	**++**	**259**	**?**	**68**	**13**	**14.5**	**mRS (30 d)**	**3.1** ${\varvec{{\text{vs}}}}$ **2.5**	**0.03**$^{a}$	**No**
Citicoline [85]		2000	++	81	?	70	12	14.3	$\Delta$Infarct volume (30 d)	18.9 ${\text{vs}}$ 11.3	0.18	No
Citicoline [84]		2001	++	899	?	68	14	13	Infarct volume (30 d)	58.9 ${\text{vs}}$ 57.7	0.80	No
Citicoline [86]		2012	++	2298	+/−	73	15	6.7	mRS $\le$1 (90 d)	18% ${\text{vs}}$ 18%	1.07 (OR) [0.85−1.36]	No
** Fasudil** [88]	Rho-kinase inhibitor	**2005**	**++**	**160**	**−**	**68**	**?**	**22**	$\Delta$**mRS** $\le$− 2 **(30 d)**	**44%** ${\varvec{{\text{vs}}}}$ **63%**	**0.0015**	**No**
Glyburide [95]	K$_{ATP}$/NC$_{Ca-ATP}$ channel inhibitor	2016	++	77	+/−	60	20	9	mRS $\le$3 (90 d)	41% ${\text{vs}}$ 39%	0.87 (OR) [0.32−2.32]$^{a}$	No
Imatinib [96]	Tyrosine kinase inhibitor	2017	+/−	60	+	73	12	4	mRS $\le$2 (90 d)	61% ${\text{vs}}$ 78%	0.296$^{a}$	No
** Sodium tanshinone IIA sulfonate** [98]	Downregulation MMP9	**2017**	**++**	**42**	**+**	**64**	**8**	**3.5**	**mRS** $\le$1 **(90 d)**	**43%** ${\varvec{{\text{vs}}}}$ **76%**	**0.028**	**?**
**Neurogenesis/-regeneration/-recovery**												
Cerebrolysin [103]	Multiple neurotrophic factors	2012	++	1067	+/−	65	9	8	mRS (90 d)	2 ${\text{vs}}$ 2	ns	No
Cerebrolysin [104]		2012	++	119	+	66	12	2	mRS $\le$1 (90 d)	55% ${\text{vs}}$ 53%	0.13 (OR) [−0.55 to 0.88]	No
** Cerebrolysin** [106]		**2017**	**++**	**100**	**−**	**68**	**10**	**?**	**mRS** $\le$1 **(30 d)**	**26%** ${\varvec{{\text{vs}}}}$ **31%**	**0.01**	**No**
** Cerebrolysin** [105]		**2016**	**++**	**40**	**+/−**	**67**	**10**	**7**	**NIHSS (21 d)**	**7.3** ${\varvec{{\text{vs}}}}$ **5.9**	<**0.05**	**No**
Choriogonadotropin + epoetin-$\alpha$[108]	Neural stem cell proliferation stimulator	2014	++	96	–	58	13	38	$\Delta$NIHSS (90 d)	− 8 ${\text{vs}}$ − 8	0.31$^{a}$	No
Cutamesine [109]	$\alpha$-1 receptor (Sig-1R) agonist	2014	++	60	+/−	67	9	59	mRS $\le$1 (56 d)	19% ${\text{vs}}$ 42%	2.68 (OR) [0.62−11.49]$^{a}$	No
Dexamphetamine [110]	TAAR1 agonist	2003	++	45	?	67	?	53	BI (90 d)	?	ns	No
** DL-3-n-butylphthalide (NBP) **[105]	Multiple neuroprotective effects	**2016**	**++**	**40**	**+/−**	**67**	**11**	**7**	**NIHSS (21 d)**	**7.3** ${\varvec{{\text{vs}}}}$ **5.5**	<**0.01**	**No**
Erythropoietin [111]	Erythropoiesis stimulator	2009	++	460	+/−	68	13	4	Lesion volume (7 d)	91 ${\varvec{{\text{vs}}}}$ 86	0.67	Yes
Filgrastim [113]	Granulocyte colony stimulating factor	2012	++	60	+/−	72	10	233	Lesion volume (90 d)	5.2 ${\varvec{{\text{vs}}}}$ 27.6	0.51	No
Filgrastim [112]		2013	++	323	+/−	69	12	7	Lesion volume (30 d)	65.9 ${\text{vs}}$ 59.0	0.44$^{a}$	No
GSK-249320 [114]	Blocking Myelin-associated glycoprotein	2017	++	134	+/−	68	10	52	mRS (90 d)	2.8 ${\text{vs}}$ 2.7	ns	No
** Kallikrein** [117]	Serine proteinase	**2011**	**++**	**44**	**?**	**68**	**10**	**42**	$\Delta$**Lesion volume (14 d)**	**0** ${\varvec{{\text{vs}}}}$ **81.5**	**0.009**	**No**
** Neurotropin** [120]	Neurotrophic factor	**1994**	**++**	**144**	**+/−**	**72**	**?**	**?**	$\Delta$**Lesion volume (11 d)**	**0.17** ${\varvec{{\text{vs}}}}$ **− 1.27**	**0.02**	**No**
**Neuroinflammation**												
Atorvastatin [19]	HMG-CoA reductase inhibitor	2011	++	81	+/−	67	6	53	Lesion volume (30 d)	280 ${\text{vs}}$ 462	0.792$^{a}$	No
Atorvastatin [127]		2011	++	62	–	75	13	7	Lesion volume (3 d)	16.2 ${\text{vs}}$ 30.4	0.33	No
Ceftriaxone [128]	Bacterial cell wall synthesis inhibitor	2015	+	2538	+/−	74	5	?	mRS $\le$2 (90 d)	60% ${\text{vs}}$ 62%; 0.94 (OR)	[0.80–1.11]	No
Cyclosporine [132]	Calcineurin inhibitor	2015	+	110	+	68	13	2.5	$\Delta$Lesion volume (30 d)	28.8 ${\text{vs}}$ 21.8	0.18	No
Enlimomab [136]	ICAM antibody	2001	++	625	–	69	15	4.5	mRS $\le$1 (90 d)	34% ${\text{vs}}$ 27%	0.004$^{a}$	Yes
** Fingolimod** [137]	S1P-R1 antagonist	**2015**	**+/−**	**47**	**+**	**59**	**12**	**3**	**Lesion volume (7 d)**	**12.1** ${\varvec{{\text{vs}}}}$ **− 2.3**	<**0.01**	**No**
** Minocycline **[139]	Microglia activation inhibiton	**2007**	**+/−**	**152**	**+/−**	**66**	**8**	**13**	**mRS (90 d)**	**2.1** ${\varvec{{\text{vs}}}}$ **0.9**	<**0.0001**	**No**
Minocycline [140]		2013	+/−	95	+/−	68	9	11	mRS $\le$2 (90 d)	33% ${\text{vs}}$ 29%	0.94 (RR) [0.71–1.25]	No
Moxifloxacin [141]	Antibiotic	2008	++	79	?	72	16	?	BI $\ge$60 (6 mo)	55% ${\text{vs}}$ 64%	0.57	No
Natalizumab [147]	$\alpha$4 integrin antagonist	2017	++	161	+/−	70	13	7	$\Delta$Lesion volume (30 d)	4 ${\text{vs}}$ 4	0.68	No
Simvastatin [148]	HMG-CoA reductase inhibitor	2008	++	60	–	73	12	?	mRS (90 d)	3 ${\text{vs}}$ 3	0.86	Yes
Simvastatin [149]		2016	++	104	+/−	74	7	7	mRS$\le$2 (90 d)	70% ${\text{vs}}$ 69%	0.99 (OR) [0.35−2.78]$^{a}$	No
UK-279,276 [151]	Neutrophil inhibitor	2003	++	746	+/−	72	13	4	Mortality (90 d)	15% ${\text{vs}}$ 17%	ns	No
**Additional compounds**												
Acetaminophen[153]	Inhibit cyclo-oxygenases or serotonerge system	2001	++	42	?	70	5	?	mRS (5 d)	?	ns	No
Acetaminophen [152]		2009	++	1400	+/−	70	7	6	mRS $\le$2 (90 d)	50% ${\text{vs}}$ 51%	1.09 (OR) [0.88−1.33]	No
** DP-b99 **[154]	Zinc and calcium ion chelator	**2008**	**++**	**150**	**–**	**73**	**12**	**6**	**mRS** $\le$1** (90 d)**	**16%** ${\varvec{{\text{vs}}}}$ **31%**	**0.05**	**No**
Reptinotan [155]	(5-HT)1A receptor agonist	2009	++	681	+/−	70	15	?	mRS $\le$2 (90 d)	38% ${\text{vs}}$ 32%	0.136$^{a}$	No

$^{\infty }$ ++ = double blinded; + = single blinded; +/− = Open-label, blinded analysis; − = no blinding

$^{\theta }$ Placebo controlled trial, Yes (Y) or No (N)

$^{\omega }$ Number of included patients (N)

$^{\dagger }$ + = (r)tPA and/or MT; +/− = “standard treatment” or mixed standard treatment and no treatment; − = No additional treatment; ? = not reported

$^{\lambda }$ Mean or median age in years (y)

$^{\rho }$ Mean or median NIHSS at baseline

$^{\xi }$ Time to treatment: (estimated) mean or median time from symptom onset to treatment (or else inclusion time); in hours (h), unless stated otherwise

$^{\pi }$ Effect size = Placebo $versus$ active treatment group ($^{a}$ = adjusted)

$^{\ddagger }$ Significance level (P) or confidence interval (CI)

NIHSS = The National Institutes of Health Stroke Scale; mRS = modified Rankin Scale; BI = Barthel Index; (m)MS = (modified) Mathew Sale; Good outcome = mRS $\le$1 or NIHSS $\le$1; ? = unknown; d = days; mo = months;

$\Delta$ = difference in outcome measure between baseline and readout timepoint; RR = Risk Ratio; OR = Odds Ratio; IVT = Intravenous trombolyses; EVT = Endovascular thrombectomy; AngII = Angiotensin II;

DCLHb = Diaspirin cross-linked hemoglobin; ACE = angiotensin-converting enzyme; CBF = cerebral blood flow; AMPA = ${\alpha }$-amino-3-hydroxy-5-methyl-4-isoxazolepropionic acid; NMDA =* N*-Methyl-d-aspartate;

GABA = ${\gamma }$-aminobutyric acid; K = Potassium; ATP = Adinosine triphosphate; NC$_{Ca}$ = nonselective cation channel for calcium; MMP9 = Matrix metallopeptidase 9; TAAR1 = Trace amine-associated receptor 1;

ICAM = intercellular adhesion molecules; HMG-CoA = ${\beta }$-Hydroxy ${\beta }$-methylglutaryl-Co-enzyme A; S1P = sfingosine 1-fosfaat; 5-HT = 5-hydroxytryptamine (serotonin); LD = low dose; HD = high dose; -R = Receptor

### Systemic haemodynamics

#### Background

Well known risk factors for ischemic stroke are abnormal (cerebral or global) haemodynamic conditions such as hypertension. Five out of 10 compounds included here are blood pressure lowering agents, since hypertension is known to accelerate the development of atherosclerosis, which in turn leads to increased atherothrombotic events [12]. Three other compounds act as vasodilators and reduce vasospasm. Prophylactic treatment to influence haemodynamic factors and reduce the (recurrent) infarct risk has been standard therapy for years. However, the influence of these same haemodynamic parameters are also likely to influence the infarct evolution and outcome when used in the acute phase of an ischemic infarct.

#### Compounds

##### Atenolol

*Atenolol* is a selective β_1_-blocking agent which is known to lower the heart rate and blood pressure. In a clinical trial including 201 AIS patients, [13] *Atenolol* (50 mg/day for 21 days) was found to reduce heartrate by 10–15% and blood pressure (the first 24 h) by 9% compared with placebo. Changes in functional outcome and neurological deficit were seen in favour of the treatment group at 30 days, but not 1 year of follow-up. Mortality, however, was more common in the *Atenolol* group at 30 days (25% vs 14%).

##### Candesartan

The angiotensin II receptor antagonist *Candesartan* was evaluated for its effect on stroke outcome in the ACCESS [14] and the SCAST trial [15–17]. In the ACCESS trial, no treatment effect (4 mg at day 1 and 8-16 mg until day 7) was seen on primary clinical outcome (BI at 30 days). And although the trial treatment goal was to accompany a reduction in blood pressure of 10–15%, no difference was found in blood pressure in the 1 year follow-up period between treatment and control group. There were no safety concerns, at the contrary, significant less cardiovascular events were reported in the Candesartan group compared with placebo. In the SCAST trial, although lower blood pressure was reported in the treatment group (147/82 mmHg vs 152/84 mmHg in the placebo group at day 7), no clinical outcome benefit (mRS or BI) of *Candesartan* (4-16 mg/day for 7 days) was found in AIS patients with high blood pressure (systolic BP ≥ 140). Importantly, a possible higher risk of poor outcome in the treatment group was reported (adjusted OR: 1.17, 95% CI 1.00–1.38) and, although non-significant, a trend towards more detrimental vascular-related events.

##### Diaspirin cross-linked hemoglobin (DCLHb)

The haemoglobin oxygen carrier *DCLHb* (dose 75–300 mg/kg/day for 3 days) was tested in a safety trial in 85 patients with AIS (40 placebo) [18]. Arterial blood pressure increased by 21 mmHg in the treatment group. Trial results showed that *DCLHb* increased the chance of poor outcome after 3 months compared with placebo (mRS 3–6 in 85% vs 51% in placebo). Other clinical outcomes (NIHSS and BI) showed similar detrimental results. Also, more deaths (23 vs 9) and overall (serious) adverse events (67 vs 22) including fatal brain and pulmonary edema, transient renal and pancreatic insufficiency, jaundice and hemoglobinuria, were reported in the intervention group.

##### Irbesartan

*Irbesartan* lowers blood pressure by acting as an angiotensin II receptor antagonist. Treatment of AIS patients with *Irbesartan* (n = 23; 150 mg/day for 30 days) did not have a significant effect on lowering the blood pressure. Neither infarct size nor clinical outcome (NIHSS) was affected by treatment [19]. Safety concerns were not addressed.

##### Lifarizine

*Lifarizine* (piperazine, Syntex) is a novel, potassium channel modulator which inhibits Na-currents in neuronal cells. It is also shown to be a calcium channel blocker and binds to the dopamine DA2 receptors. Although it has minimal direct effects on those channels it could possibly reduce BBB leakage and edema formation. In a pilot safety study among 117 AIS patients, *Lifarizine* (250 µg/kg bolus + 60 mg/day for 5 days) did not change any clinical outcome measure (mRS, NIHSS or BI at 30/90 days) [20]. Mortality or serious adverse events did not differ between study groups.

##### Lisinopril

*Lisinopril* is an angiotensin-converting enzyme (ACE) inhibitor effective for lowering systemic blood pressure. In a trial in AIS patients [21], *Lisinopril* (5 mg/day for 7 days and thereafter 10 mg/day for 7 days) or placebo was administered, starting within 24 h after stroke onset. Besides the blood pressure lowering effect no effect on clinical outcome (NIHSS, BI, mRS) was found at 14 and 90 days. There were no safety concerns.

##### Magnesium sulfate

*Magnesium sulfate* is a vasodilator, which has shown promising neuroprotective as well as gliaprotective effects in pre-clinical studies. In the IMAGES trial (included 2386 patients) [22], no effect of *Magnesium sulfate* (16 mmol bolus + 65 mmol in 24 h) on primary outcome (mRS at 90 days) was found. Mortality, however, was slightly higher in the treatment group (19% vs 16%, p = 0.086). In a second trial, the FAST-MAG trial [23], the effect of *magnesium sulfate* (20 mg in 24 h) vs*.* placebo treatment was investigated in stroke-suspected patients (n = 1700) with treatment started within 2 h after symptom onset by paramedics before hospital arrival. No effect was seen at 90 day follow-up on mRS, NIHSS, BI or GOS. Mortality or serious adverse events did not differ between study groups.

##### Nimodipine

*Nimodipine,* a calcium channel blocking agent, is widely used as treatment to reduce high blood pressure. It is also routinely used in patients with subarachnoid haemorrhage to prevent delayed cerebral ischemia. *Nimodipine* has been tested in multiple AIS trials. One of the first clinical stroke trials with *Nimodipine* was conducted by Paci et al*.* in 1989 [24]. Patients (n = 41) were treated with *Nimodipine* (40 mg/day) or placebo for 28 days, starting within 12 h after stroke onset. *Nimodipine* was found safe and well tolerated without any severe cardiovascular events. They also reported a favourable clinical outcome in the treatment group, based on the Mathew score (MS), compared with placebo (89 vs 78, respectively). A year earlier, Gelmers et al*.* [25] also reported favourable outcome for AIS patients after *Nimodipine* treatment (120 mg/day for 30 days). Better clinical outcome score (Mathew score) was reported in the treatment group, as well as decreased mortality, although the latter was restricted to the male population. Years later, the INWEST trial [26] showed a favourable outcome on BI after 21 days of treatment (1–2 mg/h for 5 days + 120 mg/day from day 5–21) compared with placebo (10 vs 22, respectively). The other 7 trials on the effect of *Nimodipine* (ranging from 60 to 240 mg/day for 10–28 days) failed to show any treatment effect concerning clinical (mRS, BI, MS or mortality) or radiological (infarct volume) outcome parameters. One trial by Kaste et al. [27], reported an increased case-fatality rate in the treatment group at 30 days (29 vs 22 in placebo). However, this difference lost statistical significance at the 1-year follow-up.

##### Piracetam

*Piracetam* is a nootropic drug with many proposed mechanisms, including allosteric AMPA receptor modulation and vascular and neuronal stimulation. However, the exact functionality is not fully understood. The PASS trial [28] reported no significant difference in BI score in the treatment group (12 g/day for 30 days + 4.8 g/day from day 31–60) compared with placebo at 90 days. Also, no significant differences in adverse events or mortality were found between the treatment and control group.

##### Propranolol

The β-blocking agent Propranolol was also tested in the BEST trial [29], next to Atenolol. A total of 201 AIS patients were included, of which half was treated with propranolol (80 mg/day for 21 days). Heart rate was reduced by 10–15% in the treatment group compared with the control group, and mean blood pressure was lowered by 6% during the first 24 h of treatment. Mortality was increased in the treatment group at 30 days (27% vs 14% in placebo) and at 6 months (33% vs 23%, respectively). Neurologic improvement and functional outcome (activity in daily living) after 1 week or 1 month was also less favourable in the treatment group compared with placebo.

### Excitotoxicity

#### Background

The brain has a high metabolic rate, requiring a large amount of oxygen and glucose to maintain the ionic gradients over the plasma membrane. The majority of the energy is consumed by the Na^+^/K^+^-ATPase of the neuronal plasma membrane. Already within the first seconds to minutes of ischemia, the ATP is fully consumed when synthesis is inhibited [30]. This results in cellular catabolic enzymes causing necrosis of cellular structures. This ionic imbalance and membrane depolarization also leads to excessive glutamate release and activation of the AMPA and NMDA receptors, resulting in increased Ca^2+^ influx. Blocking this pathway has been an attractive therapeutic target to inhibit cell death due to calcium-overload.

Other pathways contributing to excitotoxicity and eventually cell death are the metabotropic glutamate (mGlu) receptors modulating excitatory synaptic transmission [31, 32] and peri-infarct depolarizations [33]. The latter is triggered by potassium and excitatory amino acids from the ischemic core, resulting in intraneuronal calcium accumulation, cytotoxic edema and reduction in blood flow due to neurovascular coupling [33]. Excitotoxicity (disturbed ion balance) is one of the first pathways activated after vessel occlusion and therefore has been a therapeutic target in many clinical trials discussed below.

#### Compounds

##### Aptiganel HCl

*Aptiganel HCl* is an NMDA-receptor antagonist and has been tested in AIS patients in a phase II and III trial [34]. In both trials, *Aptiganel HCl* (5 mg + 0.75 mg/h or 3 mg + 0.5 mg/h for 12 h) did not show an effect on clinical outcome (mRS at 90 days or NIHSS at 7 days) compared with placebo. However, a safety-issue was noticed concerning increased mortality in the high-dose intervention group compared with placebo after a 120 days follow-up period (26% vs 19%, respectively).

##### Clomethiazole

*Clomethiazole*, an enhancer of the GABA-ergic system, has been tested in AIS patients in three consecutive trials (CLASS (n = 1360) [35], CLASS-I (n = 1198) [36] and CLASS-T (n = 190) [37]). Data of these trails all showed no difference in clinical (mRS, NIHSS or BI) or radiological (infarct volume) outcome measures between the treatment (68/75 mg/kg in 24 h, started within 12 h from onset) and the control groups. There were no differences in (serious) adverse events or mortality seen between both groups.

##### Diazepam

*Diazepam* is part of the benzodiazepine family and modulates the GABA type A receptor and acts as a calcium channel blocker. The effect of *Diazepam* on AIS has been tested in one clinical study [38] where total of 849 patients were included within 12 h of stroke onset. Diazepam (10 mg, 2 daily for 3 days) was found to be safe with no change in adverse events or mortality compared with placebo. However, no treatment effect on clinical outcome measures (mRS, BI) was found. Subgroup analyses suggested a favourable outcome in the *Diazepam* group in cardio-embolic infarct patients (mRS ≤ 2 at 90 day: OR 2.26, 95% CI [1.07–4.76]), but also an increased mortality in ICH patient population compared with placebo (22% vs 12%, respectively).

##### Fanapanel

The only competitive AMPA-receptor antagonist tested in AIS patients is *Fanapanel* (also known as ZK-200775 or MPQX). The trial was designed as a dose-finding study and included 61 AIS patients [39]. Overall neurologic (NIHSS at 30 days) worsening was seen after treatment with *Fanapanel* (262.5/525/105 mg in 6 or 48 h) compared with placebo. Due to safety reasons, this trial was prematurely stopped.

##### Gavestinel

*Gavestinel* (or GV150526) is another NMDA-receptor antagonist tested in a trial including 1367 AIS patients [40]. Treatment with *Gavestinel* (800 mg + 400 mg/24 h for 3 days) did not have an effect on clinical outcome (mRS, NIHSS, BI, infarct volume). There were no safety concerns.

##### Lanicemine HCl

*Lanicemine HCl* (or AR-R15896AR) [41], is also an NMDA-receptor antagonist and was investigated in a trial including 103 AIS patients. No beneficial effects (NIHSS, BI) were seen in the treatment group (two times a bolus of 7 mg/kg and 2.5 mg/kg + 360 mg/24 h for 3 days), compared with placebo. Safety concerns were raised due to increased mortality (10% vs 6%, respectively, non-significant) and psychiatric conditions (3 vs 0, respectively). Other side effects which were also more common in the treatment group were vomiting, nausea, fever, agitation, dizziness and hallucinations.

##### Lithium

Another NMDA-receptor antagonist tested in AIS patients is *Lithium* [42]. In a trial including 66 AIS patients, again no effect of the treatment (600 mg/24 h for 30 days) was seen on clinical outcome (NIHSS at 30 days). There were no safety concerns.

##### Lubeluzole

On the contrary, the indirect NMDA-blocking agent *Lubeluzole* did show a positive effect on clinical outcome in 1 out of 3 studies and reduced mortality was seen in 2 out of 3 studies compared with placebo. Grotta et al*.* [43] (n = 700) showed that a bolus of 7.5 mg + 10 mg/day for 5 days improved clinical outcome parameters (mRS ≤ 2 at 90 day: 36% vs 30% in placebo). Also NIHSS and BI were improved and mortality was non-significantly different in favor of the treatment group. However, Diener et al., conducted 2 trials, one including AIS patients with age ≥ 18 [44] (n = 675) and one ≥ 50 [45] (n = 193) years old. Both trials did not show a favorable outcome (mRS, NIHSS, BI) using *Lubeluzole* (7.5 mg + 10 mg/day or 15 mg + 20 mg/day for 5 days) compared with placebo. In the higher dose group, mortality was increased in the treatment group (35%), but in the lower dose it was decreased (6%) compared with placebo (18%) [45]. In the other trial of Diener et al*.* [44] no mortality differences were found between treatment and control group. However, in a post-hoc analysis, *Lubeluzole* decreased mortality without increasing morbidity in patients with mild to moderate AIS.

##### Nalmefene

*Nalmefene* (60 mg in 24 h), an opioid antagonist with relative k-receptor selectivity, has been tested in AIS patients (n = 330), starting within 6 h after stroke onset [46]. No safety concerns were seen, but also no treatment effect was shown for the clinical outcome parameters NIHSS, BI, GOS in a follow-up period until 90 days.

##### Selfotel

The last NMDA-receptor antagonist, *Selfotel* (bolus injection of 1.5 mg/kg given within 6 h after symptom onset) was tested in the ASSIST trial [47, 48]. Enrolment was prematurely stopped for safety reasons, concerning mortality was increased in the treatment group due to brain related events (progression of stroke and primarily cerebral oedema). No benefit in clinical outcome (NIHSS, BI) was found compared with placebo.

### Oxidative and nitrosative stress

#### Background

Directly after ischemic stroke onset, depletion of ATP, ion pump failure and consequently disruption in membrane ion balance occurs and eventually causing downstream production of, among others, reactive oxygen species (ROS), increased nitric oxide synthase (NOS) and nitric oxide (NO). Accumulation of these toxic compounds is most abundant in the penumbra, especially after reperfusion. Production of ROS and oxidative stress disrupts the functional BBB integrity and mediate mitochondrial and DNA damage which results in necrosis [49].

#### Compounds

##### Disufenton sodium

The effect of *Disufenton sodium* (NXY-059), a free-radical (nitrone-based) scavenger, on ischemic stroke outcome has been evaluated in multiple clinical trials, however, with different efficacy outcomes. A first safety and tolerability study [50] with *Disufenton sodium* (85/170 mg/h for 3 days) showed no safety concerns, but also no improvement in NIHSS or BI at 30 days follow-up. As a follow-up study, the SAINT I [51–53] and SAINT II [51, 54] were conducted, both using a dose of 2270 mg bolus injection in 1 h + 480–960 mg/h for 3 days. A treatment benefit was reported in the SAINT I trial (1699 patients included) at 7 days for mRS, NIHSS and BI over placebo. At 30 days, only BI and mRS still showed significant treatment benefit and at 90 days only mRS (OR 1.20. 95% CI [1.01–1.42]) was still beneficial for the treatment group. The SIANT-II trial (3306 patients included) however, was not able to confirm the results found in SAINT-I. No treatment effect for mRS, NIHSS or BI was found. Pooled analyses of both trials also failed to show any treatment benefit [51] No differences in mortality or (severe) adverse events between treatment and placebo were found.

##### Ebselen

*Ebselen* is an organoselenium compound which acts as a glutathione peroxidase mimetic and acting against membrane hydroperoxides and can potentially inhibit enzymatic and nonenzymatic lipid peroxidation, preventing cellular damage induced by reactive oxygen species (ROS) [55, 56]. The Ebselen study group conducted two trials [57, 58], and reported a favourable clinical outcome in the treatment group (150 mg/day for 14 days) compared with placebo (BI ≥ 75 at 90 days: 64% vs 50%) in the first study which included 300 patients [58]. A favourable treatment effect on GOS over placebo at 30 and 90 days was only found in patients treated within 24 h after stroke onset.

A second study in 99 patients with a MCA occlusion [57], reported a reduction in infarct volume at 30 days of follow-up in the *Ebselen* (150 mg/day for 14 days) treatment group compared with placebo (8.30 vs 12.74, respectively), an effect only significant in patients treated within 6 h after stroke onset. No differences in mortality or (serious) adverse events were seen between both treatment groups.

##### Edaravone

The free radical scavenger *Edaravone* is used in some Japanese stroke units as standard treatment. The Edaravone Acute Brain Infarction Study Group [59] showed an improvement in mRS after *Edaravone* treatment (60 mg/day for 14 days, started within 72 h after stroke onset) compared with placebo at 3, 6 and 12 months after stroke onset (n = 250). The mean mRS at 90 days was 1.8 vs 2.2 in the placebo group. No other outcome measures were described. No safety concerns were found.

##### Epigallocatechin Gallate

The effect of *Epigallocatechin Gallate*, a natural polyphenol which acts as anti-oxidant, was investigated by Wang et al*.* [60] in 371 patients. No difference was found in clinical outcome (NIHSS at 7 days) between the treatment (500 mg/day for 7 days) and placebo group. No safety data were reported.

##### Glyceryl trinitrate

Transdermal *Glyceryl trinitrate (GTN)* is a nitric oxide donor which could reduce lesion size if given in the acute phase of ischemic stroke. GTN is shown to lower the average blood pressure. However, investigators of the ENOS trial [61] (n = 4011) did not find an effect on clinical outcome at 90 days (mRS, BI) of *GTN* (5 mg/day for 7 days, started within 48 h after onset). A second study, the RIGHT-2 study (n = 852) [62], also reported no difference in *GTN* treatment (5 mg/day for 3 days) concerning mRS outcome at 90 days compared with placebo. No safety concerns in AIS patients were seen due to study medication in both trials. However, the RIGHT-2 study did report safety concerns in ultra-acute intracerebral haemorrhage patients (n = 145) who were given *GTN* (prehospital). Findings included increased mRS at 90 days (non-significant), a worse average of 5 clinical outcomes (dependency, disability, cognition, quality of life), increased mortality (in hospital, not at 90 days). Besides that, *GTN* was also associated with increased mass effect and midline shift, larger hematoma and growth and altered use of hospital resources [63]. A meta-analysis on the effect of GTN in AIS patients can be found elsewhere [64].

##### Uric acid

The natural antioxidant *uric acid* (UA) has been studied in AIS in the URICO-ICTUS trial [65–69]. *UA* (1000 mg in 90 min) treatment showed overall favourable clinical outcome over placebo (mRS at 90 days: 2 vs 3, respectively, p = 0.045). Both BI and NIHSS also showed favourable outcomes at 90 days, but no differences in ASPECTS score or infarct volume were found between treatment and control group. In pre-defined sub-analyses (n = 135–138 patients per group), improved clinical outcome and limited infarct growth was only found in *UA* treated patients with high glucose levels (upper tertile range). Better clinical results were found in *UA* treated woman (n = 206), but not in men (n = 205) (pre-defined subgroup analysis). No safety concerns were found.

##### Tirilazad Mesylate

The effect of the lipid peroxidation inhibitor and scavenger of free radicals, *Tirilazad,* was investigated in two clinical trials. The STIPAS trial [70] (n = 111) showed that treatment of maximal 6 mg/kg/day for 3 days of *Tirilazad Mesylate* was safe. No difference between the treatment and placebo group were found concerning adverse events or mortality. Although the study was not designed to find treatment effects, they reported no difference in infarct volume. Concerning clinical outcome scores, the placebo group showed worse outcome parameters defined by GOS (favourable outcome with 2 mg/kg/day: 85% vs 92% in placebo group, p = 0.028). Both BI and NIHSS showed the same trend.

Thereafter, 4 clinical trials (RANTTAS I and II [71] and TESS I and II [72]) were conducted using the highest dose (6 mg/kg/day for 3 days). The RANTTAS trials (556 AIS patients) were stopped prematurely to allow for inclusion of another trial with higher *Tirilazad* dose (decision based upon interim analyses, but also studies in SAH patients and pre-clinical data. No favourable effect on clinical outcome (NIHSS, BI or GOS at 3 months) of the treatment over placebo was found. There were no safety concerns. In the TESS-I and II trials [72], CT was used to assess the effect of *Tirilazad Mesylate* on infarct outcome on day 8 (± 2 days) in 368 patients. Again no effect on clinical or radiological outcome was seen. Only in male patients with a cortical infarct, treatment with *Tirilazad* showed significant smaller infarct volumes (pre-defined subgroup (n = 136) analysis: 71 mL vs 143 mL in placebo, p = 0.001).

A review and meta-analysis of these clinical trials conducted for the effect of *Tirilazad Mesylate* in AIS even showed an increase in death or disability in the *Tirilazad* treatment group [73].

### Blood-brain barrier dysfunction and vasogenic edema

#### Background

Blood–brain barrier (BBB) disruption following ischemic stroke is an early pathological event resulting in extravasation of blood components into the brain parenchyma. Disruption of this functional and physical barrier results in multiple detrimental effects, such as vasogenic edema formation, excessive cellular and molecular infiltration into the brain parenchyma including many inflammatory cells and cytokines, disrupting the interstitial composition, haemorrhagic transformation and eventually exacerbating brain injury [49, 74, 75]. During cerebral ischemia, one important contribution to BBB breakdown is matrix metalloproteinase (MMP) activation, followed by integrin (transmembrane glycoprotein receptors for the extracellular matrix) degradation.

#### Compounds

##### Albumin

The effect of A*lbumin* (the main blood plasma protein regulating oncotic pressure) in AIS patients have extensively been investigated. The ALIAS trials (pilot [76], I [77–80] and II [77, 79]) used a 2-h infusion of 25% human albumin (within 5 h (part I) or within 16 h (part II) of stroke onset). In the ALIAS part I trial, no difference in NIHSS or mRS at 90 days were found. The trial was terminated after 434 patients were enrolled, for safety reasons due to higher 90-day mortality rate and increased serious adverse events (a.o. pulmonary edema, acute coronary syndrome and myocardial infarction) in the *Albumin* group. ALIAS part II (with modified study protocol) included 841 patients when it was prematurely stopped for futility reasons. No difference in NIHSS, mRS or BI at 90 days were found between the treatment and control group. More adverse events (mild-to-moderate pulmonary edema and symptomatic intracranial haemorrhage) were reported in the *Albumin*-treated patients.

##### Citicoline

*Citicoline* is an intermediate compound in the turnover of choline into phosphatidylcholine, a process naturally occurring in cell the membrane. In preclinical experiments, it has been shown that C*iticoline* (CDP-choline) can reduce cell membrane breakdown, increase phosphatidylcholine synthesis and decrease free fatty acids [81, 82]. The effect of C*iticoline* in AIS has been investigated in multiple clinical trials. Clarck et al*.* [83] reported a favourable clinical outcome (mRS at 30 days: 2.5 vs 3.1 in placebo, p = 0.03) in the treatment group both for 500 and 2000 mg/day for 6 weeks. The same positive effect was seen in BI and NIHSS at 90 days after AIS in the C*iticoline* treatment group compare with placebo. However, in a follow-up trial Clark et al*.* [84] reported no effect of C*iticoline* (200 mg/day for 6 weeks) at 90 days concerning lesion volume (on MRI), NIHSS, mRS and BI. Warach et al*.* [85] also investigated the effect of C*iticoline* (500 mg/day for 6 weeks) on ischemic lesion evolution using diffusion MRI (measured from baseline to 12 weeks). Although a difference in lesion evolution (favouring *Citicoline*; + 11.3 vs + 18.9 in placebo) was seen between treatment and control group, variance was too high and power too low to reach statistical significance. There was however a significant larger reduction in infarct volume from week 1 to week 12 reported in the treatment group. Davalos et al. [86] investigated a slightly higher dose (2000 mg/day for 3 days, where after 1000 mg/day for 6 weeks) of C*iticoline* in a total of 1148 AIS patients compared with 1150 placebo treated AIS patients. No treatment benefits were seen here concerning mRS, NIHSS or BI at 90 days. The trial was prematurely stopped for futility reasons. No difference in (serious) adverse events were found in all above mentioned studies. “A comprehensive review and meta-analysis concerning *Citicoline* can be found elsewhere [87]”.

##### Fasudil

*Fasudil*, a Rho-kinase inhibitor, is a vasodilator and preserves BBB integrity by blocking endothelial and pericyte contraction. It has shown efficacy in subarachnoid haemorrhage patients to reduce vasospasm [88]. In pre-clinical studies, *Fasudil* was showed an anti-inflammatory effect [89], prevent monocyte and neutrophil infiltration and inhibit the production of oxygen radicals [90], increase cerebral blood flow [91] and upregulate eNOS activity of endothelial cells [92]. In a trial including 160 AIS patients [93], *Fasudil* (60 mg/60 min twice daily, for 14 days) improved mRS at 30 days over placebo (improvement of mRS ≤ − 2: 63% vs 44% in the placebo group). No safety issues were found.

##### Glyburide

*Glyburide* is a compound generally used to treat diabetes mellitus type 2. It is a K_ATP_/NC_Ca-ATP_ channel inhibitor by binding to the subunit sulfonylurea receptor 1 (SUR1). It is also known for its antioxidant effects. The effect of G*lyburide* (Glibenclamide, RP-1127) in AIS was tested in the GAMES-Pilot [94] (< 15 patients/group) and GAMES-PR [95] trials. In the GAMES-PR trial, the effect of *Glyburide* (0.13 mg bolus + 0.16 mg/h (6 h) + 0.11 mg/h (66 h)) in 77 patients was analysed. Although the trial was prematurely stopped due to financial reasons, a decreased 30-day mortality rate was found in the treatment group compared with placebo (15% vs 36%, respectively, p = 0.03). This effect could be attributed to decrease in malignant edema. BBB-specific outcomes in post hoc analysis of edema-related endpoints also showed a 50% reduction of midline shift and plasma MMP-9 levels in the treatment group. No difference in mRS at 90 days was found between both groups. No safety issues were identified.

##### Imatinib

*Imatinib* is a tyrosine kinase inhibitor which could have a beneficiary effect on maintaining BBB integrity and therefore reduce haemorrhagic transformation, edema formation and infarct size. *Imatinib* was tested in a randomized phase II trial at three dose levels (400, 600 and 800 mg for 6 days) [96]. *Imatinib* significantly improved NIHSS compared with controls during 90 days *post* stroke onset (improvement of 0.6 on the NIHSS per 100 mg *Imatinib*, p = 0.02). The same (non-significant) trend was found on mRS at 90 days. BBB-specific secondary endpoints were haemorrhagic transformation (39%, 58%, 62% and 43% in the control and low-, medium- and high-dose groups respectively) and cerebral oedema (50%, 50%, 62% and 71%, respectively), however, no statistics were described. No safety concerns were found.

##### Sodium tanshinone IIA sulfonate

*Sodium tanshinone IIA sulfonate (STS)* has been used in multiple (pre-) clinical experiments and trials, and has been said to have multiple protective effects against; BBB disruption, ischemia–reperfusion injury, and has anti-oxidative, anti-apoptotic, anti-inflammatory effects and acts as vasodilatator [97].

In 16 AIS patients, *STS* significantly improved the 90-day mRS compared with 14 placebo-treated patients (mRS ≤ 1: 76% vs 43%, respectively, p = 0.028) [98]. However, no difference in other clinical outcome scales (NIHSS or ADL) was found between treatment and control group. BBB damage was measured using biomarkers in plasma, including levels of MMP-2, MMP-9, tissue inhibitor of metalloproteinase (TIMP)-1, claudin-5 and zonula occludens (ZO)-1. Claudin-5 and MMP-9 were lower and TIMP-1 higher in the treatment group. There was also a difference found in BBB permeability on CTP at 10 days, but not 24 h, *post* rt-PA treatment between both treatment groups. Nothing was reported concerning any (serious) adverse events.

### Neurogenesis/-regeneration and -recovery

#### Background

As a response to ischemia, multiple recovery cascades become activated, including adult neurogenesis and regeneration (extensively reviewed by Marques et al*.* [99]). Neuronal precursor cells will respond by proliferation and migration towards the lesion site. In the adult brain, three neurogenic zones are identified: the subventricular zone in the lateral ventricle, subgranular zone of the dentate gyrus and third the posterior periventricular zone [100–102]. Although endogenous neurogenesis is a slow process and is not able to fully restore brain function, therapeutic strategies activating and accelerating these processes might be beneficial in AIS, as pre-clinical data is suggesting.

#### Compounds

##### Cerebrolysin

*Cerebrolysin* is a neurotrophic and neuroprotective drug consisting of low-molecular-weight neuropeptides and free amino acids of porcine origin. The CASTA trial [103] treated 529 patients with 30 mL *Cerebrolysin* daily for 10 days and compared results with 541 placebo-treated patients. Overall, no treatment effect was found for mRS, NIHSS or BI. Also no difference in mortality was found between both groups. Lang et al*.* [104] also studied the effect of *Cerebrolysin* (30 mL/day, for 10 days). However, after the third interim analysis, the study was terminated because there was no indication for favourable treatment effect at 90 days, measured by mRS, NIHSS, BI or GOS.

The safety and efficacy of *Cerebrolysin* (30 ml/day, for 10 days) vs placebo was described by Xue et al*.* [105] No safety issues were found and a favourable outcome for NIHSS at 21 days (5.9 vs 7.3 in placebo, p < 0.05) was shown. BI showed essentially the same results. A fourth trial by Gharagozli et al*.* [106] evaluated the effect of *Cerebrolysin* treatment (30 mL/day for 7 days followed by 10 mL until day 30) vs placebo. They were able to show a superiority of *Cerebrolysin* treatment compared with placebo for 30-day (mRS ≤ 1: 31% vs 26% in placebo, p = 0.01). The same positive effect was shown for NIHSS and clinical global impression. No differences were found for mini mental state examination or patient global satisfaction. None of the trials found any safety concerns for the treatment with *Cerebrolysin*. A meta-analysis concerning the effect of *Cerebrolysin* in AIS patients can be found elsewhere [107].

##### Choriogonadotropin

*Human Choriogonadotropin* (hCG) is known as the pregnancy hormone and was shown to stimulate endogenous neural stem cell proliferation in preclinical experiments. In the REGENESIS-LED trial [108], the effect of hCG (385 mg hCG (day 1 + 3 + 5) was tested in AIS patients in combination with epoetin alfa (4000–20.000 IU/dose (day 7–9)), a compound which stimulates neuronal stem cell survival and differentiation. The study was halted early because of lack of treatment benefit at 30 and 90 days (NIHSS, mRS, BI). No differences were found in any (serious) adverse events or mortality.

##### Cutamesine

*Cutamesine (SA 4503*; 1 or 3 mg/day, for 28 days*),* a sigma-1 receptor agonist, was tested in a Phase II safety trial by Urfer et al*. *[109] No serious adverse treatment events were found, also no treatment effect for clinical outcome was seen measured using NIHSS, mRS and BI.

##### Dexamphetamine

The safety of *Dexamphetamine*, a central noradrenergic stimulus, was tested in AIS patients in a dose-escalation trial [110]. Patients were given 2.5, 5 or 10 mg *Dexamphetamine* or placebo twice daily for 5 days, starting within 72 h post stroke onset. No (serious) adverse events or increased mortality corresponding to treatment were found. Multiple motor function tests were performed, but no differences were seen between treatment and placebo at follow-up (1 or 3 months).

##### DL-3-n-butylphthalide

The effect of *DL-3-n-butylphthalide (NBP)* (50 mg/day for 10 days) in AIS patients was tested in the same trial where the effect of Cerebrolysin was investigated [105]. NBP showed a favourable outcome over placebo treatment concerning NIHSS at 21 days (5.5 vs 7.3, respectively, p < 0.01). The same effect was seen for BI. Besides that, *NBP* showed to be more effective over Cerebrolysin. No safety concerns were seen for the use of *NBP*.

##### Erythropoietin

Recombinant Human *Erythropoietin (EPO)* has, besides its well-known haematopoietic effects, been said to be neuroprotective, having anti-apoptotic, anti-oxidant and anti-inflammatory effects. The effect of EPO (40.000 IU over 30 min, *iv*) within 6 h after stroke onset was evaluated by Ehrenreich et al*.* [111]. No treatment effect was seen for 30 or 90 days concerning mRS, NIHSS or BI. Also no differences between treatment and placebo groups were found on lesion characteristics using MRI. The trial did report safety concerns due to increased mortality in the *EPO* group (16.4% vs 9.0%), especially in patients who received systemic thrombolytic therapy.

##### Filgrastim

The effect of *Filgrastim* (G-CSF, AX200), a granulocyte colony-stimulating factor, was studied in a phase IIb trial of 522 AIS patients [112]. A dose of 135 µg/kg, *iv* over 72 h was analysed for its effect on stroke outcome. Due to absence of clinical (mRS, BI, NIHSS, mortality) and radiological (infarct volume) effects at 30 days *post* stroke, the trial was stopped prematurely. No differences in adverse events between treatment and control groups were found. A second trial was conducted by England et al*.* [113]. Here, a dose of 10 µg/kg *sc* was given for 5 days, starting 3–30 days post stroke symptom onset. No difference between treatment and placebo groups were found in clinical outcome (mRS, BI, NIHSS) at 90 days nor for lesion volume (MRI). No differences in (serious) adverse events between both study groups were reported.

##### GSK-249320

The humanized monoclonal antibody *GSK249320* blocks myelin-associated glycoprotein to promote axon outgrowth. Cramer et al*.* [114] conducted a phase IIb proof-of-concept trail, which was stopped prematurely due to futility reasons. No treatment benefit (a bolus of 15 mg/kg) was found during interim analysis for functional outcome (mRS). No safety concerns were found.

##### Kallikrein

*Kallikrein* (DM-199) is a serine proteinase which selectively dilates arterioles, and which has been shown in the pre-clinical setting to stimulate angiogenesis and neurogenesis [115, 116]. In a clinical trial conducted by Wang et al. [117], AIS patients received *Kallikrein* (0.15PANU/day for 12–14 days), starting within 6–72 h of infarct onset. At 30 days, *Kallikrein* showed improved mRS, although the median was equal compared with placebo (3 vs 3, p = 0.001). This effect was gone at 90 days follow-up. The same result was seen for NIHSS and BI. However, no treatment effect on infarct volume was seen at 14 days. They also found more micro-bleeds in the infarcted region in the treatment group compared with placebo, but this did not worsen any outcome measure.

##### Neurotropin

*Neurotropin (NTP)*, widely used in Japan as analgesic drug, is a non-protein extract which is isolated from cutaneous tissue of rabbits who are inoculated with vaccinia virus. NTP activates the descending pain inhibition system and is involved in anti-nociception [118, 119]. De Reuck et al. [120] described a decreased infarct volume (difference between day 3 and 11 using CT: − 1.27 vs 0.17, respectively, p = 0.02) in the treatment group compared with placebo. Also less edema and improved clinical outcome (Toronto stroke scale, from day 5 till 15) was shown. Mortality and (serious) adverse events were equal in both study groups.

### Neuro-inflammation

#### Background

The inflammatory response after cerebral vessel occlusion is multifaceted and differentiates over time from initiation and progression to resolution of the infarct. The importance of the inflammatory cascades is reflected by the many compounds which have been tested in (pre-) clinical trials, to modulate both the neurotoxic as well as the neuroprotective effects of the inflammatory response.

The inflammatory response includes both the innate and adaptive components of the immune system. The first one starts in blood vessels and the perivascular space, where hypoxia changes endothelial shear stress, triggering the coagulation cascade, inducing platelet aggravation and oxidative stress. This results in release of inflammatory mediators, BBB permeability and leukocyte/neutrophil infiltration into the infarcted area [121]. After this initial process, the brain parenchyma also gets involved in the inflammation cascade where neuronal cell death triggers a new phase of the inflammatory response. The adaptive immune response is activated somewhat later, starting around 24 h *post* occlusion [122]. The recruited T-cells have their damaging effect, exacerbating neurotoxicity by secretion of several cytokines. Later in the chronic phase (first days after onset), specific T-cell-subsets become involved such as the more damaging (Th1 or 17) or protective (Th2 and Treg) T-cells [123]. B-cell recruitment (also days after stroke onset) causes further neuronal damage [124]. Notwithstanding, T-cell, monocyte-derived macrophages/activated microglia and neutrophils all have their pro- and anti-inflammatory phenotypes, controlling a delicate balance.

The end of the initial inflammatory cascades marks itself by (1) removal of death tissue/debris, (2) development of an anti-inflammatory environment and (3) production of survival-favourable mediators [125]. The chronic inflammatory response (lasting weeks to months), a phenomenon called stroke-induced immunodepression (SIID), starts after a few days [126], but is beyond the scope of this review.

Many treatments described below target the innate immune response and are therefore restricted to the acute stroke phase. Instead, drugs modulating the adaptive immune response might be feasible in a longer timeframe.

### Compounds

#### Atorvastatin

*Statins*, HMG-CoA reductase inhibitors, belong to the class of lipid-lowering agents and are generally used preventative to reduce the risk for cardiovascular events. However, statins also have anti-inflammatory effects, an effect which was tested in the acute phase after ischemic stroke in multiple clinical trials. *Atorvastatin* was investigated in 2 randomized clinical AIS trials. Muscari et al*.* [127] did not find a difference in NIHSS (at day 7) or infarct volume on CT (at 3 days) between AIS patients given *Atorvastatin* (31 patients; 80 mg/day for 7 days) or placebo (31 patients). Nevertheless, in less severe strokes (n = 9), the 3-month mRS outcome suggested a more favourable outcome in the statin-treated group (n = 7) (Not mentioned as pre-defined subgroup analysis, number needed to treat = 3). In the second trial, Beer et al. [19] included 40 AIS patients, treated with *Atorvastatin* (80 mg/day for 30 days) or placebo. No treatment effect on radiological (infarct volume) or clinical (NIHSS, CBF) outcome was seen at 3 or 30 days after stroke onset. No safety concerns were found in both trials.

#### Ceftriaxone

*Ceftriaxone* is a third generation antibiotic compound which belongs to the cephalosporin family. It selectively binds to transpeptidases located on the bacterial cell wall and inhibits cellular wall synthesis. In the PASS trial [128], the effect of *Ceftriaxone* (2 gr/day for 4 days) over placebo was investigated in 2538 AIS patients. No difference in clinical outcome (mRS and mortality at 90 days) was found between treatment and control group. No differences in (serious) adverse events were seen between both groups.

#### Cyclosporine

*Cyclosporine* is an immunosuppressant by reducing effector T-cell function via binding to the cytosolic protein cyclophilin. It is also a potent inhibitor of the mitochondrial permeability transition pore (MPTP) and the P-glycoprotein (P-gp). MPTP allows molecular equilibration of small molecules (< 1599 Da) such as Ca^2+^ during reperfusion and P-gp is a drug resistance transporter highly expressed in the BBB. *Cyclosporine* has been shown to be effective in pre-clinical experiments to reduce ischemic damage in various organs including heart [129] and brain [130, 131]. It was tested by Nighoghossian et al*.* [132] in a pilot trial in 127 AIS patients. *Cyclosporine* was shown to reduce infarct size, but only in the sub-population were recanalization was established after proximal occlusion (pre-defined subgroup (n = 32; underpowered) analysis: 14.9 vs 48.3 respectively, p = 0.009). No overall difference was seen after 3 months in neurologic independence score (mRS) between treatment and control group. No difference in (serious) adverse events were found.

#### Enlimomab

This murine monoclonal antibody binds to the intracellular adhesion molecule-1 (ICAM-1), thereby inhibiting neutrophil adhesion to the endothelium. Although in pre-clinical studies *Enlimomab* was shown to have a positive effect on neurological function and infarct volume [133–135], this was not the case in a 90-day clinical AIS trial [136]. *Enlimomab* (160 mg *iv* bolus following 40 mg maintenance bolus/day for 4 days) even showed a significant worsened clinical outcome (mRS ≤ 1 at 90 day: 27% vs 34% respectively, p = 0.004) compared with placebo. Symptom-free recovery, serious adverse events, death, BI and NIHSS showed similar results. Infarct volume did not show a difference between both treatment groups.

#### Fingolimod

*Fingolimod* (FTY720; 0.5 mg/day for 3 days) was tested in combination with alteplase in a clinical safety trial [137]. *Fingolimod* is largely used as multiple sclerosis treatment and acts on the sphingosine-1-phosphate receptor, inhibiting lymphocyte egress from lymph nodes and inhibiting their recirculation [138]. They were able to show a significant favourable effect of the combination therapy compared with alteplase alone for infarct volume difference from day 1 to 7 (-2.3 vs 12.1 respectively, p < 0.01), although the sample was small. They also found less haemorrhage and better mRS and NIHSS scores at 90 days post AIS in the treatment group compared with placebo. No difference in adverse events were seen.

#### Minocycline

*Minocycline* is an antibiotic agent, inhibiting microglia activation and matrix metalloproteinase (MMP). The effect of *Minocycline* in AIS patients has been investigated in 2 different trials, all using different dosages. Lampl et al. [139] investigated the effect of 200 mg minocycline for 5 days, starting within 6–24 h after stroke onset in 152 patients. Clinical outcome at 7, 30 and 90 days, analyzed by NIHSS, mRS and BI, was significantly improved in the *Minocycline* treated group compared with the control group (90-day mRS: 0.9 vs 2.1 respectively, p < 0.0001). No radiological outcomes were reported. A second (pilot) study was conducted by Kohler et al. [140] and included 47 patients in the *Minocycline* (500 mg in 12 h) group and 48 controls. No differences were found concerning clinical outcome (mRS, NIHSS, BI). There were no differences between both treatment groups concerning (serious) adverse events in all three trials.

#### Moxifloxacin

*Moxifloxacin* is also an anti-bacterial compound, and prevents separation of the bacterial DNA (and thereby inhibiting cell replication) by inhibiting DNA gyrase (topoisomerase II and IV). The PANTHERIS trial [141] examined the effect of preventive antibacterial therapy in 39 AIS patients compared with 40 controls using *Moxifloxacin* (400 mg/day for 5 days). Besides the positive effect of *Moxifloxacin* treatment on reduction of post-stroke infections, the survival rate and neurological outcome defined by BI and NIHSS were not different between treatment and control group. No safety concerns were raised.

#### Natalizumab

*Natalizumab*, a humanized monoclonal antibody against the α4-integrin within very late antigen (VLA)-4, impairs trans-endothelial migration of leukocytes by preventing their interaction with endothelial cell adhesion molecule VCAM. In multiple pre-clinical studies, *Natalizumab* showed reduced infarct volume and improvement of functional outcome [142–146]. In the phase II ACTION trial [147], 161 patients were randomly assigned to placebo or 300 mg (in 24 h) *Natalizumab*. No changes in infarct volume or relative growth ratio (from baseline to 30 day follow-up) between both groups were found. At 30 day of the follow-up, an improved mRS (pre-defined subgroup analysis: mRS ≤ 1: 18% vs 9% respectively, p = 0.024) was found in patients with baseline mRS ≤ 1 in the treatment group (n = 77) compared with placebo (n = 82), but not for BI or NIHSS. However, at 90 days, only an improved outcome was seen for BI in patients with a baseline BI score ≥ 95 (pre-defined subgroup analysis in 77 *Natalizumab* and 82 placebo treated patients). No differences were found concerning (serious) adverse events between both groups.

#### Simvastatin

The effect of *Simvastatin*, member of the statin-family and an HMG-CoA reductase inhibitor, in the acute phase of ischemic stroke was investigated in two RCTs; MISTIC [148] (safety and efficacy pilot trial) and STARS [149]. In the MISTIC trial, no clinical outcome improvement (90 day mRS) of the *Simvastatin* (40 mg/day for 7 days + 20 mg/day for 83 days) group compared with placebo was found. Importantly, safety concerns were described since a (non-significant) higher mortality and infection rate was seen in these patients. The STARS trial also reported no difference between the treatment and placebo group for mRS at 90 days (n = 34 simvastatin (40 mg/day for 90 days) and 34 placebo). Post-hoc analysis did however show favourable clinical outcome for S*imvastatin* in combination with tPA (not a pre-defined subgroup analysis). The STARS trail results did not show any safety concerns.

#### UK-279,276

*UK-279,276* is a neutrophil inhibitory factor and pre-clinical data showed improvement of infarct volume after *UK-279,276* treatment in transient, but not permanent, middle cerebral artery occlusion [150]. In the ASTIN phase-2 trial [151], *UK-279,276* was examined concerning dose–response and proof-of-concept. A total of 966 AIS patients were enrolled in this study before it was terminated early for futility reasons. The compound was well tolerated, but no (dose–response; 10–120 mg in 24 h) treatment effect was seen compared with placebo for NIHSS, BI or mRS at 90 days. There were no safety issues.

### Additional compounds

#### Acetaminophen

The phase III PAIS trial [152] investigated the effect of *Acetaminophen* (6 g/day for 3 days), one of the most commonly used antipyretic drug, on AIS outcome. No treatment effect was seen in the primary analysis (mRS or BI at 90 days). However, in patients with body temperature between 37–39 °C and patients not treated with alteplase showed an improved 3-month mRS, but not BI, after pre-defined post-hoc analysis. A pilot trial conducted by Koennecke et al*.* [153] tested the effect of *Acetaminophen* (4 g/day for 5 days) on AIS outcome, started within 24 h after stroke onset. No differences between treatment and placebo groups were found concerning NIHSS or mRS at 5 days. No safety concerns were seen in both trials.

#### DP-b99

*DP-b99* is a chelator for excessive divalent metal ions (such as zinc and calcium) which is specially designed to chelate such ions in the vicinity of membranes when their concentration exceeds the normal physiological level. In the mRECT trial (150 AIS patients) [154], the effect of *DP-b99* (1 mg/kg/day for 3 days) was investigated. A significant better mRS score at 90 days was found in the treatment group compared with placebo (mRS ≤ 1 or same as prestrike: 31% vs 16%, respectively, p = 0.05). But no difference in NIHSS was found. No mortality differences or (serious) adverse events were found related to the treatment.

#### Reptinotan

*Reptinotan HCl* is a serotonin (5-HT)_1A_ receptor antagonist and the effect on AIS outcome was tested in the mRECT phase IIb trial [155]. *Reptinotan* (1 mg/kg/day or placebo for 4 days) was given within 4.5 h post stroke onset. No differences between groups were found for clinical outcome parameters (mRS, NIHSS, BI at 90 days). No safety concerns were found related to the use of *Reptinotan.*

## Discussion

In this review, we have summarized clinical AIS trials where, besides reperfusion treatment (thrombolysis or thrombectomy), additional therapeutic drugs were tested in order to improve clinical and/or radiological outcome. Only a handful of compounds were found in some way beneficial concerning main clinical or radiological outcome, mostly found in post-hoc subgroup analysis. In the few cases where multiple trials are conducted for one compound, usually no treatment effect or contradicting treatment effects were found between trials. Many trials included here were designed as safety and tolerability-trial, and therefore results should be interpreted with caution due to the low inclusion numbers.

### Categorical drug effects

Important to state here, before discussing any possible group effect, is that compounds were grouped considering their assumed (principal) mechanism of action. However, often these compounds have myriads of pharmacological (primary or secondary) effects, one stronger or weaker over the other. Besides that, it is even possible multiple drug effects could have influenced one another for the best or worst (amplifying or cancelling out one another when looking at final outcome parameters).

Looking at group effect, it seems that drugs intervening with oxidative and nitrosative stress are overall safe, well tolerated and show the most promising results (positive effect on outcome in 3/7 compounds). An interesting point here is the difference in standard stroke care around the world, regarding *Edaravone*. *Edaravone* has never reached other parts of the world, while in Japan it has been used in daily clinical stroke care since 2001. A number of clinical trials (few showed clinical benefit), meta-analyses (with positive outcomes) [156–159] and pre-clinical studies have been undertaken (all extensively reviewed elsewhere [160–162]), however, the majority were executed in Asian countries. Besides that, despite the described beneficial effect of *Edaravone*, it was also noted that these results might have been biased [163]. One practical issue was the relatively long Japanese study protocol with twice-daily intravenous treatment for up to 14 days, where in Europe, AIS hospitalization periods are usually much shorter, making the Japanese protocol impractical. Therefore, Kaste et al. [164] performed a safety, tolerability, and pharmacokinetics study, involving a new *Edaravone* formulation and dosing regimen more suitable for Europe. Their results paved the way for the necessary larger safety studies and pivotal RCTs to provide evidence of efficacy. These trials are necessary to show whether the treatment improves the outcome of patients with AIS in Western countries. Also, the use of *Edaravone* combined with thrombolysis and thrombectomy should be evaluated. However, until today this has not been initiated yet, perhaps for economic reasons since the compound patent period has already expired. *Edaravone* is also used for adult myotrophic lateral sclerosis (ALS) treatment and the many ongoing clinical trials for neurological diseases as well as multiple other pathologies and clinical conditions indicates its broad neuroprotective antioxidant possibilities [165]. So, especially in the MT-era, *Edaravone* could be a good candidate for re-evaluation as adjunctive AIS treatment.

Another promising group effect is represented by compounds effecting neurogenesis/-regeneration and -recovery. Here, 4 out of 10 compounds showed positive outcome scores in the treatment groups compared with placebo. Interestingly, these trials with positive outcome all had follow-up time points of less than 30 days. All other trials who did not show any treatment effect, except for 1 [106], had a readout time point of ≤ 21 days. This might indicate that this kind of treatment is only beneficial in the early time period after ischemic stroke, but does not last in the late chronic phase. A combination therapy with compounds focussed on the chronic effects such as inflammation might be a promising strategy. Surprisingly, most drugs suppressing neuro-inflammation do not seem to improve stroke outcome when given in the acute phase, since only 2 out of 10 compounds (*Fingolimod* and *Minocycline*) reported a favourable outcome in the treatment group over placebo. Reason for this could be that the inflammation response in the infarcted area, is bifacial; part of the inflammatory cascades are involved in increased damage and edema formation whereas other inflammatory pathways are important for neuroprotection, regeneration and restoration [166]. Therefore, roughly blocking all inflammatory processes which are activated in the acute or chronic phase after ischemic stroke, may not be a good strategy. Instead, a more targeted approach blocking detrimental inflammatory cascades and stimulating favourable ones might provide better clinical results.

Targeting systemic haemodynamics alone is also not a promising strategy (also previously shown in a meta-analyse of blood pressure lowering agents by Sandset et al. [16]) and appears even to have detrimental effects (found in 5/21 trials and shown in a sub-group analyse of the INWEST trial [167]). However, the link between blood pressure changes and clinical outcome in AIS patients is extremity complex and optimal blood pressure management remains challenging since it depends on treatment strategy, local vascular function and especially the degree of reperfusion or persistent occlusion (reviewed by Gasecki et al. [168]). Only for one highly investigated compound, *Nimodipine*, 3 out of 10 trials showed a beneficial effect when given within 24 h of stroke onset. And in a meta-analysis, data of more than 3700 AIS patients supported the view that early treatment with *Nimodipine* may have a favourable effect [169]. However, interestingly, the 6 trials where *Nimodipine* was given within 48 h all did not show any treatment effect. The last one also did not show an effect, although treatment was given within 6 h. This also proves that the time window of treatment is a crucial but complex factor in AIS therapy.

Care should be taken with compounds described in the excitotoxicity category, especially NMDA receptor antagonists, but also AMPS receptor antagonists. In 5 out of 10 compounds (5/14 trials) included here (4/8 NMDA-receptor antagonists), safety concerns were reported when administering the antagonist in the acute phase of an ischemic stroke. Only one (out of 3) trial (Grotta et al. [43]) reported an improved mRS, NIHSS and BI after treatment with *Lubeluzole,* and 2/3 trials showed reduced mortality*.* The positive effect was only seen in low-dose treatment of *Lubeluzole*, where a dose twice as high had an unfavourable outcome. Therefore, one could argue that the negative and worsened outcomes found in the other NMDA-receptor studies could be because the used doses were too high. Outcome using *Lubeluzole* might also be more favourable due to the fact that is an indirect NMDA-blocking agent [170].

### Patient sub-group effects

An interesting finding is that due to the large variability in ischemic stroke patients, the mean outcome effects were usually non-existing. However, in post-hoc analyses in specific sub-populations, multiple trials did find a positive drug effect on outcome. This was for example seen in the *Candesartan* trial, where a beneficial effect on mRS was reported in a subset of patients with large infarcts compared with small, lacunar infarcts. And a negative effect for BI was reported in the *Candesartan* group in a subset of patients with lacunar infarcts [16, 17]. Another example is *Piracetam*, for which only in a *post-hoc* analyses including early treated patients or patients with moderate and severe stroke, a difference in clinical outcome favouring the treatment group was found [28].

This raises the question if these secondary results are actual solid evidence of stroke category-specific treatment effects, or if this is a false positive treatment effect due to multiple testing. If the first is true, this would mean that in order to find most effective therapies, the focus should be pointed more towards better patient stratification and a more personalized therapeutic approach concerning sex, stroke etiology or reperfusion rate for example.

In addition, worth mentioning is that clinical outcome variables mostly used in stroke trials such as mRS, NIHSS and BI, not always correspond with one another and even less with radiological outcome measures such as infarct volume or diffusion and perfusion abnormalities. Maybe a better patient stratification and sub-group specific scales or treatment could be a step towards improved AIS care [171].

### Combination therapies and EVT/IVT

The number 1 question concerning additional drug therapeutics (next to IVT and EVT) in AIS is whether or not the drug reaches the infarcted territory sufficiently. Multiple clinical studies examining recanalization as well as tissue reperfusion concluded that reperfusion was essential to achieve good functional recovery. Good reperfusion of the tissue was a 4 times stronger predictor of final outcome compared to recanalization of the vessel or collateral status [172–174]. Interestingly, reperfusion via collaterals was also associated with improved clinical outcome even without recanalization of the occluded vessel [175].

Unfortunately, in almost all trials included here, patients were included irrespective of recanalization treatment (none, IVT or EVT, or a combination), different therapeutic time windows, or reperfusion rate. All these factors are likely to affect the outcomes of adjuvant treatments. The introduction of endovascular treatment for AIS has revolutionized stroke care. While mechanical thrombectomy with stent retrievers or aspiration devices has become the standard of care nowadays [4, 176, 177], various interventional techniques have been tried over the years. Early trials using intra-arterial pro-urokinase showed promising results, but did report high rates of bleeding [178]. Subsequent clinical trials that mostly used intra-arterial tPA or earlier generation thrombectomy devices, and which did not routinely use CT-angiography to select patients with a large vessel occlusions, showed neutral results [179–181]. After the initial successful trials with modern EVT techniques, subsequent trials expanded the time window for EVT until 24 h for patients with salvageable brain tissue on perfusion imaging [182, 183]. The recent developments in AIS treatment warrant a reconsideration of the neuroprotective strategies, either because they might show to be more effective after recanalization, or, in contrast, their value becomes less in the patients that receive late EVT.

Post-hoc analysis of the effect of the compound on outcome compared between patients who received recanalization therapy (and their reperfusion rate) and who did not, is necessary to investigate if the compound could be given a second chance in combination with thrombectomy. Besides that, without the knowledge and sub-analyses concerning the correlation of reperfusion rate and clinical outcome, it is hard to conclude if the drug itself did not have an effect. Interaction between reperfusion and outcome could be due to the fact that a sufficient amount of the drug did not reach the area of interest (in time, due to no or unsatisfactory reperfusion of the smaller arteries and microvasculature) or due to the presumed mechanism of action of the specific compound, namely reperfusion injury. It is well possible that additional drug treatment effect is overshadowed by this heterogeneity in patient population. A good example of this is the *Cyclosporine* trial [132], where they did analyze the effect of recanalization therapy and showed the compound to reduce infarct size, but only in the sub-population were recanalization was established after proximal occlusion.

Drugs with promising results in combination with thrombolytic therapy might even be more beneficial in combination with the new, more superior endovascular treatment. This possibility should be investigated in newly designed clinical trials. Also more strict time window could help increasing the therapeutic success rate. Time window is one of the most crucial factors in acute stroke treatment to increase efficacy, and intervening in more than one of the ischemic cascades could reduce detrimental effects or increase the effective therapeutic time window which is also a valuable improvement.

Therefore, new trials with promising drugs in combination with reperfusion therapy such as mechanical thrombectomy (if successful) is promising.

Also because many of these compounds have been shown effective in pre-clinical studies in mice and rats where usually the transient middle cerebral artery occlusion is used. (Meaning, reperfusion of the MCA after a certain period of time). This ischemic model is different form the heterogeneous AIS patient group which usually do not have (sufficient) reperfusion of the occluded artery. Other known factors contributing to the translational gap are the pre-clinical models used, design of the clinical trials, clinical misclassification, restricted therapeutic window and inadequate sample size [171, 184, 185]. This translational gap, however outside the scope of this review, is an important topic to consider, and which is emphasized by the positive pre-clinical data in mice and rats, but the negative clinical trial results of the same compounds. As stroke researchers, we should think critically which fundamental issues are the cause of this lack of translational value.

Another important concern influencing the success rate and efficacy, especially in AIS treatment, is the pharmacokinetic properties of a compound. Molecular size, polarity and hydrophobicity, each affect the ability of the compound to be transferred across the BBB and reach the target area. *Disufenton sodium* (NXY-059) for instance, failed in multiple clinical trials [50–54], which might have been caused by the fact that *disufenton sodium* is a polar molecule with low hydrophobicity, which limits its ability to permeate the BBB [186, 187]. Therefore, it is of utmost importance to consider drug pharmacokinetic properties during therapeutic development, and investigate how their molecular characteristics can affect efficacy in stroke patients. One way to manage this problem are the multiple targeted drug delivery systems which have been developed over the years, including the use of (polymer/metal/gold) nanoparticles, exosomes or structural modification of the therapeutic compounds. A detailed overview concerning current techniques and possibilities can be found elsewhere [188].

The unfortunate bulk of negative findings summarized in this review seems contradictive compared to the pre-clinical evidence of effectiveness of the compounds described here. This discrepancy originates from the methodological and translational limitations of pre-clinical stroke research, pre-clinical publication bias (negative study results are hardly ever published) but also the lack of (meta-analyses supported) pre-clinical evidence of the effectiveness of the compounds when being selected for a clinical trial [189]. Besides that, the disappointing trial results are probably also caused by the complex multi-faced string of events activated the moment a cerebral artery is occluded. The complex interplay of systemic haemodynamics, excitotoxicity, neuro-inflammation, blood–brain barrier and vasogenic edema, oxidative and nitrosative stress all eventually leads to cell death. All clinical trials described in this review focus to intervene in one (small) part or one of the mechanisms, and looking at the negative results together with the complexity of the activated cascades, one might conclude that trying to improve outcome after AIS is not possible when only one aspect is targeted using a single agent. Together with the large heterogeneity of stroke patients (concerning important parameters such as type of occluded vessel, age, sex, race and other cardiovascular risk factors and comorbidities), it may be unrealistic to expect general improved outcome using only a single neuroprotective drug. Therefore, future stroke trials should focus on a combination therapies in order to increase treatment benefits for AIS patients. Combinatorial therapy in AIS is probably the most promising therapeutic strategy and should be considered when designing new clinical trials. Important to add here is the therapeutic options to “freeze the penumbra” before reperfusion therapy can be performed to prevent infarct growth in the first hours after onset [190] (instead of only starting the additional treatment afterwards, as done in all clinical trials so far).

There are a number of clinical trials (registered and/or recruiting) testing additional therapeutic compounds specifically in combination with mechanical thrombectomy. These include (registered at ClinTrials.gov June 2021): Pulmozyme (NCT04785066); combination of argatroban, edaravone, and glucocorticoid (INSIST-CT, NCT04202549); Tirofiban (NCT04851457); Fingolimod (NCT04675762 and NCT04629872); Cerebrolysin (NCT04904341); Edaravone Dexborneol (INSIST-ED, NCT04667637); Nerinetide (ESCAPE-NEXT, NCT04462536); Verapamil (NCT03347786); Butylphthalide (NCT03539445); RNS60 (NCT04693715); P2Y12 inhibitor (Cangrelor) (NCT04667078) and multiple blood pressure controlling compounds (both during and in the acute phase after MT) (NCT04205305, ENCHANTED2 NCT04140110, NCT04352296, NCT04892511 and NCT04578288). It is to be seen whether or not these combination therapies are beneficial for patient outcome.

### Future perspectives

Ischemic stroke is an extremely complex, acute pathology with a large variety of pathways involved in neuronal damage and repair, all with their own time window making the best-treatment puzzle hard to solve. Therefore, we do not believe that clinical improvement can be accomplished by treating all AIS patients with only a single drug class. In our opinion, focus should be on (1) investigating the underlying pathophysiologic changes in individual stroke sub-types; (2) Identifying pathways and therapeutic targets with respect to timing after stroke onset and (3) Composing a treatment strategy for each stroke-subtype, targeting multiple pathways, each with its own dosage and time-dependant regime. In line with the data summarized in this review, reperfusion therapy (EVT/IVT) in combination with oxidative and nitrosative stress inhibitors (such as Ebselen, Edaravone, Glyceryl Trinitrate or Uric acid), vascular modifiers (Fasudil) and targeted anti-inflammatory agents (such as Cyclosporine, Fingolimod or Minocycline) could be a promising strategy in the (hyper) acute phase, with subsequent promotion of resolution of inflammation and neurorecovery in the late-acute and chronic phase. Some animal studies have shown the positive effect of combination-therapy in ischemic stroke [191–194].

## Conclusion

There is an enormous collection of clinical AIS trials with negative outcome, however, one should not be discouraged by that. The most promising drugs for additional treatment combined with EVT are oxidative and nitrosative stress inhibitors and promotors of neurogenesis/-regeneration and -recovery. The least promising (or even dangerous) drugs seem to be NMDA and AMPA receptor blocking agents and compounds targeting systemic haemodynamics. Care should be taken concerning crucial parameters for future clinical AIS trials: start of the treatment, duration of the treatment, and combination of thrombolysis and/or thrombectomy with additional drugs to intervene with detrimental ischemia-related pathways and stimulate regenerating pathways with the superiority of endovascular thrombectomy over standard medical care [4], new possibilities are there since reperfusion in a crucial factor in order to deliver the drug towards the region of interest (penumbra and core).

## Data Availability

Data sharing not applicable to this article as no datasets were generated or analyzed during the current study.

## Abbreviations

5-HT: 5-Hydroxytryptamine (Serotonin)
ACE: Angiotensin-converting enzyme
AIS: Acute ischemic stroke
AMPA: α-Amino-3-hydroxy-5-methyl-4-isoxazolepropionic acid
ATP: Adenosin triphosphate
BBB: Blood-brain barrier
BI: Barthel Index
CBF: Cerebral blood flow
CI: Confidence interval
CT(P): Computed tomography (perfusion)
DCLHb: Diaspirin cross-linked hemoglobin
EPO: Erythropoietin
EVT: Endovascular thrombectomy
GOS: Glasgow Outcome Score
GTN: Glyceryl trinitrate
hCG: Human choriogonadotropin
ICAM-1: Intracellular adhesion molecule-1
Iv: Intravenous
IVT: Intravenous thrombolysis
MCA: Middel cerebral artery
MMP: Matrix metalloproteinase
MPTP: Mitochondrial permeability transition pore
MRI: Magnetic resonance imaging
mRS: Modified Rankin Scale
NBP: DL-3-*n*-butylphthalide
NIHSS: National Institutes of Health Stroke Scale
NMDA: *N*-Methyl-d-aspartic acid
NO(S): Nitric oxide (synthase)
NTP: Neurotropin
OR: Odds ratio
P-gp: P-glycoprotein
RCT: Rondomized controlled trial
ROS: Reactive oxygen species
STS: Sodium tanshinone IIA sulfonate
TIMP: Tissue inhibitor of metalloproteinase
tPA: Tissue plasminogen activator
UA: Uric acid
VCAM: Vascular cell adhesion molecule
VLA: Very late antigen
Vs: Versus
ZO: Zonula occludens

## References

[1] National Institute of Neurological D, Stroke rt PASSG. Tissue plasminogen activator for acute ischemic stroke. N Engl J Med. 1995;333:1581–7. 10.1056/NEJM199512143332401 7477192

[2] Macrae IM, Allan SM (2018). Stroke: the past, present and future. Brain Neurosci Adv.

[3] Berkhemer OA, Fransen PS, Beumer D, van den Berg LA, Lingsma HF, Yoo AJ (2015). A randomized trial of intraarterial treatment for acute ischemic stroke. N Engl J Med.

[4] Goyal M, Menon BK, van Zwam WH, Dippel DW, Mitchell PJ, Demchuk AM (2016). Endovascular thrombectomy after large-vessel ischaemic stroke: a meta-analysis of individual patient data from five randomised trials. Lancet.

[5] Sweid A, Hammoud B, Ramesh S, Wong D, Alexander TD, Weinberg JH (2020). Acute ischaemic stroke interventions: large vessel occlusion and beyond. Stroke Vasc Neurol.

[6] Savitz SI, Baron JC, Yenari MA, Sanossian N, Fisher M (2017). Reconsidering neuroprotection in the reperfusion era. Stroke.

[7] Savitz SI, Baron JC, Fisher M, Consortium SX (2019). Stroke treatment academic industry roundtable x: Brain cytoprotection therapies in the reperfusion era. Stroke.

[8] Shea BJ, Grimshaw JM, Wells GA, Boers M, Andersson N, Hamel C (2007). Development of amstar: a measurement tool to assess the methodological quality of systematic reviews. BMC Med Res Methodol.

[9] Moher D, Liberati A, Tetzlaff J, Altman DG, Group P (2009). Preferred reporting items for systematic reviews and meta-analyses: the prisma statement. BMJ.

[10] Goslinga H, Eijzenbach V, Heuvelmans JH, van der Laan de Vries E, Melis VM, Schmid-Schonbein H (1992). Custom-tailored hemodilution with albumin and crystalloids in acute ischemic stroke. Stroke.

[11] Harrison JK, McArthur KS, Quinn TJ (2013). Assessment scales in stroke: clinimetric and clinical considerations. Clin Interv Aging.

[12] Sacco RL, Adams R, Albers G, Alberts MJ, Benavente O, Furie K (2006). Guidelines for prevention of stroke in patients with ischemic stroke or transient ischemic attack: a statement for healthcare professionals from the American heart association/American stroke association council on stroke: co-sponsored by the council on cardiovascular radiology and intervention: the American academy of neurology affirms the value of this guideline. Stroke.

[13] Barer DH, Cruickshank JM, Ebrahim SB, Mitchell JR (1988). Low dose beta blockade in acute stroke (“best” trial): an evaluation. Br Med J (Clin Res Ed).

[14] Schrader J, Luders S, Kulschewski A, Berger J, Zidek W, Treib J (2003). The access study: evaluation of acute candesartan cilexetil therapy in stroke survivors. Stroke.

[15] Hornslien AG, Sandset EC, Wyller TB, Berge E, Scandinavian Candesartan Acute Stroke Trial Study G (2015). Effects of candesartan in acute stroke on activities of daily living and level of care at 6 months. J Hypertens.

[16] Sandset EC, Bath PM, Boysen G, Jatuzis D, Korv J, Luders S (2011). The angiotensin-receptor blocker candesartan for treatment of acute stroke (scast): a randomised, placebo-controlled, double-blind trial. Lancet.

[17] Sandset EC, Jusufovic M, Sandset PM, Bath PM, Berge E, Group SS (2015). Effects of blood pressure-lowering treatment in different subtypes of acute ischemic stroke. Stroke.

[18] Saxena R, Wijnhoud AD, Carton H, Hacke W, Kaste M, Przybelski RJ (1999). Controlled safety study of a hemoglobin-based oxygen carrier, dclhb, in acute ischemic stroke. Stroke.

[19] Beer C, Blacker D, Bynevelt M, Hankey GJ, Puddey IB (2012). A randomized placebo controlled trial of early treatment of acute ischemic stroke with atorvastatin and irbesartan. Int J Stroke.

[20] Squire IB, Lees KR, Pryse-Phillips W, Kertesz A, Bamford J (1995). Efficacy and tolerability of lifarizine in acute ischemic stroke. A pilot study. Lifarizine study group. Ann N Y Acad Sci.

[21] Eveson DJ, Robinson TG, Potter JF (2007). Lisinopril for the treatment of hypertension within the first 24 hours of acute ischemic stroke and follow-up. Am J Hypertens.

[22] Muir KW, Lees KR, Ford I, Davis S (2004). Intravenous Magnesium Efficacy in Stroke Study I. Magnesium for acute stroke (intravenous magnesium efficacy in stroke trial): randomised controlled trial. Lancet.

[23] Saver JL, Starkman S, Eckstein M, Stratton SJ, Pratt FD, Hamilton S (2015). Prehospital use of magnesium sulfate as neuroprotection in acute stroke. N Engl J Med.

[24] Paci A, Ottaviano P, Trenta A, Iannone G, De Santis L, Lancia G (1989). Nimodipine in acute ischemic stroke: a double-blind controlled study. Acta Neurol Scand.

[25] Gelmers HJ, Gorter K, de Weerdt CJ, Wiezer HJ (1988). A controlled trial of nimodipine in acute ischemic stroke. N Engl J Med.

[26] Wahlgren NG, Macmahon DG, Dekeyser J, Indredavik B, Ryman T (1994). Intravenous nimodipine west-European stroke trial (inwest) of nimodipine in the treatment of acute ischemic stroke. Cerebrovasc Dis.

[27] Kaste M, Fogelholm R, Erila T, Palomaki H, Murros K, Rissanen A (1994). A randomized, double-blind, placebo-controlled trial of nimodipine in acute ischemic hemispheric stroke. Stroke.

[28] De Deyn PP, Reuck JD, Deberdt W, Vlietinck R, Orgogozo JM (1997). Treatment of acute ischemic stroke with piracetam. Members of the piracetam in acute stroke study (pass) group. Stroke.

[29] Barer Dh CJ, Ebrahim SB, Mitchell JRA (1988). Low dose blockade in acute stroke (“best” trial): an evaluation. BMJ.

[30] Doyle KP, Simon RP, Stenzel-Poore MP (2008). Mechanisms of ischemic brain damage. Neuropharmacology.

[31] Bruno V, Battaglia G, Copani A, D'Onofrio M, Di Iorio P, De Blasi A (2001). Metabotropic glutamate receptor subtypes as targets for neuroprotective drugs. J Cereb Blood Flow Metab.

[32] Pellegrini-Giampietro DE (2003). The distinct role of mglu1 receptors in post-ischemic neuronal death. Trends Pharmacol Sci.

[33] Hartings JA, Shuttleworth CW, Kirov SA, Ayata C, Hinzman JM, Foreman B (2017). The continuum of spreading depolarizations in acute cortical lesion development: examining leao’s legacy. J Cereb Blood Flow Metab.

[34] Albers GW, Goldstein LB, Hall D, Lesko LM (2001). Aptiganel Acute Stroke I. Aptiganel hydrochloride in acute ischemic stroke: a randomized controlled trial. JAMA.

[35] Wahlgren NG, Ranasinha KW, Rosolacci T, Franke CL, van Erven PM, Ashwood T (1999). Clomethiazole acute stroke study (class): results of a randomized, controlled trial of clomethiazole versus placebo in 1360 acute stroke patients. Stroke.

[36] Lyden P, Shuaib A, Ng K, Levin K, Atkinson RP, Rajput A (2002). Clomethiazole acute stroke study in ischemic stroke (class-i): final results. Stroke.

[37] Lyden P, Jacoby M, Schim J, Albers G, Mazzeo P, Ashwood T (2001). The clomethiazole acute stroke study in tissue-type plasminogen activator-treated stroke (class-t): final results. Neurology.

[38] Lodder J, van Raak L, Hilton A, Hardy E, Kessels A (2006). Diazepam to improve acute stroke outcome: results of the early gaba-ergic activation study in stroke trial. A randomized double-blind placebo-controlled trial. Cerebrovasc Dis.

[39] Elting JW, Sulter GA, Kaste M, Lees KR, Diener HC, Hommel M (2002). Ampa antagonist zk200775 in patients with acute ischemic stroke: possible glial cell toxicity detected by monitoring of s-100b serum levels. Stroke.

[40] Sacco RL, DeRosa JT, Haley EC, Levin B, Ordronneau P, Phillips SJ (2001). Glycine antagonist in neuroprotection for patients with acute stroke: gain Americas: a randomized controlled trial. JAMA.

[41] Diener HC, AlKhedr A, Busse O, Hacke W, Zingmark PH, Jonsson N (2002). Treatment of acute ischaemic stroke with the low-affinity, use-dependent nmda antagonist ar-r15896ar. A safety and tolerability study. J Neurol.

[42] Mohammadianinejad SE, Majdinasab N, Sajedi SA, Abdollahi F, Moqaddam MM, Sadr F (2014). The effect of lithium in post-stroke motor recovery: a double-blind, placebo-controlled, randomized clinical trial. Clin Neuropharmacol.

[43] Grotta J (1997). Lubeluzole treatment of acute ischemic stroke. The us and canadian lubeluzole ischemic stroke study group. Stroke.

[44] Diener HC (1998). Multinational randomised controlled trial of lubeluzole in acute ischaemic stroke. European and Australian lubeluzole ischaemic stroke study group. Cerebrovasc Dis.

[45] Diener HC, Hacke W, Hennerici M, Radberg J, Hantson L, De Keyser J (1996). Lubeluzole in acute ischemic stroke. A double-blind, placebo-controlled phase ii trial. Lubeluzole international study group. Stroke.

[46] Clark WM, Raps EC, Tong DC, Kelly RE (2000). Cervene (nalmefene) in acute ischemic stroke: final results of a phase iii efficacy study. The cervene stroke study investigators. Stroke.

[47] Davis SM, Albers GW, Diener HC, Lees KR, Norris J (1997). Termination of acute stroke studies involving selfotel treatment. Assist steering committed. Lancet.

[48] Davis SM, Lees KR, Albers GW, Diener HC, Markabi S, Karlsson G (2000). Selfotel in acute ischemic stroke: possible neurotoxic effects of an nmda antagonist. Stroke.

[49] Abdullahi W, Tripathi D, Ronaldson PT (2018). Blood–brain barrier dysfunction in ischemic stroke: TARGETING tight junctions and transporters for vascular protection. Am J Physiol Cell Physiol.

[50] Lees KR, Sharma AK, Barer D, Ford GA, Kostulas V, Cheng YF (2001). Tolerability and pharmacokinetics of the nitrone nxy-059 in patients with acute stroke. Stroke.

[51] Diener HC, Lees KR, Lyden P, Grotta J, Davalos A, Davis SM (2008). Nxy-059 for the treatment of acute stroke: pooled analysis of the saint i and ii trials. Stroke.

[52] Lees KR, Davalos A, Davis SM, Diener HC, Grotta J, Lyden P (2006). Additional outcomes and subgroup analyses of nxy-059 for acute ischemic stroke in the saint i trial. Stroke.

[53] Lees KR, Zivin JA, Ashwood T, Davalos A, Davis SM, Diener HC (2006). Nxy-059 for acute ischemic stroke. N Engl J Med.

[54] Shuaib A, Lees KR, Lyden P, Grotta J, Davalos A, Davis SM (2007). Nxy-059 for the treatment of acute ischemic stroke. N Engl J Med.

[55] Muller A, Cadenas E, Graf P, Sies H (1984). A novel biologically active seleno-organic compound-i. Glutathione peroxidase-like activity in vitro and antioxidant capacity of pz 51 (ebselen). Biochem Pharmacol.

[56] Wendel A, Fausel M, Safayhi H, Tiegs G, Otter R (1984). A novel biologically active seleno-organic compound-ii. Activity of pz 51 in relation to glutathione peroxidase. Biochem Pharmacol.

[57] Ogawa A, Yoshimoto T, Kikuchi H, Sano K, Saito I, Yamaguchi T (1999). Ebselen in acute middle cerebral artery occlusion: a placebo-controlled, double-blind clinical trial. Cerebrovasc Dis.

[58] Yamaguchi T, Sano K, Takakura K, Saito I, Shinohara Y, Asano T (1998). Ebselen in acute ischemic stroke: a placebo-controlled, double-blind clinical trial. Ebselen Study Group. Stroke.

[59] Edaravone Acute Infarction Study G (2003). Effect of a novel free radical scavenger, edaravone (mci-186), on acute brain infarction. Randomized, placebo-controlled, double-blind study at multicenters. Cerebrovasc Dis.

[60] Wang XH, You YP (2017). Epigallocatechin gallate extends therapeutic window of recombinant tissue plasminogen activator treatment for brain ischemic stroke: a randomized double-blind and placebo-controlled trial. Clin Neuropharmacol.

[61] Investigators ET (2015). Efficacy of nitric oxide, with or without continuing antihypertensive treatment, for management of high blood pressure in acute stroke (enos): a partial-factorial randomised controlled trial. Lancet.

[62] Investigators R (2019). Prehospital transdermal glyceryl trinitrate in patients with ultra-acute presumed stroke (right-2): an ambulance-based, randomised, sham-controlled, blinded, phase 3 trial. Lancet.

[63] Bath PM, Woodhouse LJ, Krishnan K, Appleton JP, Anderson CS, Berge E (2019). Prehospital transdermal glyceryl trinitrate for ultra-acute intracerebral hemorrhage: data from the right-2 trial. Stroke.

[64] Bath PM, Woodhouse L, Krishnan K, Anderson C, Berge E, Ford GA (2016). Effect of treatment delay, stroke type, and thrombolysis on the effect of glyceryl trinitrate, a nitric oxide donor, on outcome after acute stroke: a systematic review and meta-analysis of individual patient from randomised trials. Stroke Res Treat.

[65] Amaro S, Llull L, Renu A, Laredo C, Perez B, Vila E (2015). Uric acid improves glucose-driven oxidative stress in human ischemic stroke. Ann Neurol.

[66] Amaro S, Laredo C, Renu A, Llull L, Rudilosso S, Obach V (2016). Uric acid therapy prevents early ischemic stroke progression: a tertiary analysis of the urico-ictus trial (efficacy study of combined treatment with uric acid and r-tpa in acute ischemic stroke). Stroke.

[67] Chamorro A, Amaro S, Castellanos M, Gomis M, Urra X, Blasco J (2017). Uric acid therapy improves the outcomes of stroke patients treated with intravenous tissue plasminogen activator and mechanical thrombectomy. Int J Stroke.

[68] Chamorro A, Amaro S, Castellanos M, Segura T, Arenillas J, Marti-Fabregas J (2014). Safety and efficacy of uric acid in patients with acute stroke (urico-ictus): a randomised, double-blind phase 2b/3 trial. Lancet Neurol.

[69] Llull L, Laredo C, Renu A, Perez B, Vila E, Obach V (2015). Uric acid therapy improves clinical outcome in women with acute ischemic stroke. Stroke.

[70] Investigators S (1994). Safety study of tirilazad mesylate in patients with acute ischemic stroke (stipas). Stroke.

[71] Scott P, Barsan W, Frederiksen S, Kronick S, Zink BJ, Domeier RM (1996). A randomized trial of tirilazad mesylate in patients with acute stroke (ranttas). Stroke.

[72] van der Worp HB, Kappelle LJ, Algra A, Bar PR, Orgogozo JM, Ringelstein EB (2002). The effect of tirilazad mesylate on infarct volume of patients with acute ischemic stroke. Neurology.

[73] Tirilazad mesylate in acute ischemic stroke (2000). A systematic review. Tirilazad international steering committee. Stroke.

[74] DiNapoli VA, Huber JD, Houser K, Li X, Rosen CL (2008). Early disruptions of the blood-brain barrier may contribute to exacerbated neuronal damage and prolonged functional recovery following stroke in aged rats. Neurobiol Aging.

[75] Yang Y, Rosenberg GA (2011). Blood-brain barrier breakdown in acute and chronic cerebrovascular disease. Stroke.

[76] Palesch YY, Hill MD, Ryckborst KJ, Tamariz D, Ginsberg MD (2006). The alias pilot trial: a dose-escalation and safety study of albumin therapy for acute ischemic stroke–ii: neurologic outcome and efficacy analysis. Stroke.

[77] Ginsberg MD, Palesch YY, Hill MD, Martin RH, Moy CS, Barsan WG (2013). High-dose albumin treatment for acute ischaemic stroke (alias) part 2: a randomised, double-blind, phase 3, placebo-controlled trial. Lancet Neurol.

[78] Hill MD, Martin RH, Palesch YY, Tamariz D, Waldman BD, Ryckborst KJ (2011). The albumin in acute stroke part 1 trial: an exploratory efficacy analysis. Stroke.

[79] Martin RH, Yeatts SD, Hill MD, Moy CS, Ginsberg MD, Palesch YY (2016). Alias (albumin in acute ischemic stroke) trials: analysis of the combined data from parts 1 and 2. Stroke.

[80] Ginsberg MD, Palesch YY, Martin RH, Hill MD, Moy CS, Waldman BD (2011). The albumin in acute stroke (alias) multicenter clinical trial: safety analysis of part 1 and rationale and design of part 2. Stroke.

[81] Agut J, Lopez GCI, Ortiz JA, Wurtman RJ (1993). Oral cytidine 5′-diphosphate choline administration to rats increases brain phospholipid levels. Ann N Y Acad Sci.

[82] Knapp S, Wurtman RJ (1999). Enhancement of free fatty acid incorporation into phospholipids by choline plus cytidine. Brain Res.

[83] Clark WM, Warach SJ, Pettigrew LC, Gammans RE, Sabounjian LA (1997). A randomized dose-response trial of citicoline in acute ischemic stroke patients. Citicoline stroke study group. Neurology.

[84] Clark WM, Wechsler LR, Sabounjian LA, Schwiderski UE, Citicoline Stroke Study G (2001). A phase iii randomized efficacy trial of 2000 mg citicoline in acute ischemic stroke patients. Neurology.

[85] Warach S, Pettigrew LC, Dashe JF, Pullicino P, Lefkowitz DM, Sabounjian L (2000). Effect of citicoline on ischemic lesions as measured by diffusion-weighted magnetic resonance imaging. Citicoline 010 investigators. Ann Neurol.

[86] Davalos A, Alvarez-Sabin J, Castillo J, Diez-Tejedor E, Ferro J, Martinez-Vila E (2012). Citicoline in the treatment of acute ischaemic stroke: an international, randomised, multicentre, placebo-controlled study (ictus trial). Lancet.

[87] Marti-Carvajal AJ, Valli C, Marti-Amarista CE, Sola I, Marti-Fabregas J, Bonfill CX (2020). Citicoline for treating people with acute ischemic stroke. Cochrane Database Syst Rev.

[88] Shibuya M, Suzuki Y, Sugita K, Saito I, Sasaki T, Takakura K (1992). Effect of at877 on cerebral vasospasm after aneurysmal subarachnoid haemorrhage. Results of a prospective placebo-controlled double-blind trial. J Neurosurg.

[89] Satoh S, Utsunomiya T, Tsurui K, Kobayashi T, Ikegaki I, Sasaki Y (2001). Pharmacological profile of hydroxy fasudil as a selective rho kinase inhibitor on ischemic brain damage. Life Sci.

[90] Arai M, Sasaki Y, Nozawa R (1993). Inhibition by the protein kinase inhibitor ha1077 of the activation of nadph oxidase in human neutrophils. Biochem Pharmacol.

[91] Nagata K, Kondoh Y, Satoh Y, Watahiki Y, Yokoyama E, Yuya H (1993). Effects of fasudil hydrochloride on cerebral blood flow in patients with chronic cerebral infarction. Clin Neuropharmacol.

[92] Takemoto M, Sun J, Hiroki J, Shimokawa H, Liao JK (2002). Rho-kinase mediates hypoxia-induced downregulation of endothelial nitric oxide synthase. Circulation.

[93] Shibuya M, Hirai S, Seto M, Satoh S, Ohtomo E, Fasudil Ischemic Stroke Study G (2005). Effects of fasudil in acute ischemic stroke: results of a prospective placebo-controlled double-blind trial. J Neurol Sci.

[94] Kimberly WT, Battey TW, Pham L, Wu O, Yoo AJ, Furie KL (2014). Glyburide is associated with attenuated vasogenic edema in stroke patients. Neurocrit Care.

[95] Sheth KN, Elm JJ, Molyneaux BJ, Hinson H, Beslow LA, Sze GK (2016). Safety and efficacy of intravenous glyburide on brain swelling after large hemispheric infarction (games-rp): a randomised, double-blind, placebo-controlled phase 2 trial. Lancet Neurol.

[96] Wahlgren N, Thoren M, Hojeberg B, Kall TB, Laska AC, Sjostrand C (2017). Randomized assessment of imatinib in patients with acute ischaemic stroke treated with intravenous thrombolysis. J Intern Med.

[97] Fu J, Huang H, Liu J, Pi R, Chen J, Liu P (2007). Tanshinone iia protects cardiac myocytes against oxidative stress-triggered damage and apoptosis. Eur J Pharmacol.

[98] Ji B, Zhou F, Han L, Yang J, Fan H, Li S (2017). Sodium tanshinone iia sulfonate enhances effectiveness rt-pa treatment in acute ischemic stroke patients associated with ameliorating blood-brain barrier damage. Transl Stroke Res.

[99] Marques BL, Carvalho GA, Freitas EMM, Chiareli RA, Barbosa TG, Di Araujo AGP (2019). The role of neurogenesis in neurorepair after ischemic stroke. Semin Cell Dev Biol.

[100] Eriksson PS, Perfilieva E, Bjork-Eriksson T, Alborn AM, Nordborg C, Peterson DA (1998). Neurogenesis in the adult human hippocampus. Nat Med.

[101] Gage FH, Kempermann G, Palmer TD, Peterson DA, Ray J (1998). Multipotent progenitor cells in the adult dentate gyrus. J Neurobiol.

[102] Vieira MS, Santos AK, Vasconcellos R, Goulart VAM, Parreira RC, Kihara AH (2018). Neural stem cell differentiation into mature neurons: mechanisms of regulation and biotechnological applications. Biotechnol Adv.

[103] Heiss WD, Brainin M, Bornstein NM, Tuomilehto J, Hong Z (2012). Cerebrolysin Acute Stroke Treatment in Asia I. Cerebrolysin in patients with acute ischemic stroke in Asia: results of a double-blind, placebo-controlled randomized trial. Stroke.

[104] Lang W, Stadler CH, Poljakovic Z, Fleet D, Lyse Study G (2013). A prospective, randomized, placebo-controlled, double-blind trial about safety and efficacy of combined treatment with alteplase (rt-pa) and cerebrolysin in acute ischaemic hemispheric stroke. Int J Stroke.

[105] Xue LX, Zhang T, Zhao YW, Geng Z, Chen JJ, Chen H (2016). Efficacy and safety comparison of dl-3-n-butylphthalide and cerebrolysin: effects on neurological and behavioral outcomes in acute ischemic stroke. Exp Ther Med.

[106] Gharagozli K, Harandi AA, Houshmand S, Akbari N, Muresanu DF, Vester J (2017). Efficacy and safety of cerebrolysin treatment in early recovery after acute ischemic stroke: a randomized, placebo-controlled, double-blinded, multicenter clinical trial. J Med Life.

[107] Ziganshina LE, Abakumova T, Hoyle CH (2020). Cerebrolysin for acute ischaemic stroke. Cochrane Database Syst Rev.

[108] Cramer SC, Hill MD, Investigators R-L (2014). Human choriogonadotropin and epoetin alfa in acute ischemic stroke patients (regenesis-led trial). Int J Stroke.

[109] Urfer R, Moebius HJ, Skoloudik D, Santamarina E, Sato W, Mita S (2014). Phase ii trial of the sigma-1 receptor agonist cutamesine (sa4503) for recovery enhancement after acute ischemic stroke. Stroke.

[110] Martinsson L, Wahlgren NG (2003). Safety of dexamphetamine in acute ischemic stroke: a randomized, double-blind, controlled dose-escalation trial. Stroke.

[111] Ehrenreich H, Weissenborn K, Prange H, Schneider D, Weimar C, Wartenberg K (2009). Recombinant human erythropoietin in the treatment of acute ischemic stroke. Stroke.

[112] Ringelstein EB, Thijs V, Norrving B, Chamorro A, Aichner F, Grond M (2013). Granulocyte colony-stimulating factor in patients with acute ischemic stroke results of the ax200 for ischemic stroke trial. Stroke.

[113] England TJ, Abaei M, Auer DP, Lowe J, Jones DR, Sare G (2012). Granulocyte-colony stimulating factor for mobilizing bone marrow stem cells in subacute stroke: the stem cell trial of recovery enhancement after stroke 2 randomized controlled trial. Stroke.

[114] Cramer SC, Enney LA, Russell CK, Simeoni M, Thompson TR (2017). Proof-of-concept randomized trial of the monoclonal antibody gsk249320 versus placebo in stroke patients. Stroke.

[115] Ling L, Hou Q, Xing S, Yu J, Pei Z, Zeng J (2008). Exogenous kallikrein enhances neurogenesis and angiogenesis in the subventricular zone and the peri-infarction region and improves neurological function after focal cortical infarction in hypertensive rats. Brain Res.

[116] Xia CF, Yin H, Yao YY, Borlongan CV, Chao L, Chao J (2006). Kallikrein protects against ischemic stroke by inhibiting apoptosis and inflammation and promoting angiogenesis and neurogenesis. Hum Gene Ther.

[117] Wang YD, Lu RY, Huang XX, Yuan F, Hu T, Peng Y (2011). Human tissue kallikrein promoted activation of the ipsilesional sensorimotor cortex after acute cerebral infarction. Eur Neurol.

[118] Kawai H, Asaoka N, Miyake T, Nagayasu K, Nakagawa T, Shirakawa H (2018). Neurotropin inhibits neuronal activity through potentiation of sustained kv currents in primary cultured drg neurons. J Pharmacol Sci.

[119] Miura T, Okazaki R, Yoshida H, Namba H, Okai H, Kawamura M (2005). Mechanisms of analgesic action of neurotropin on chronic pain in adjuvant-induced arthritic rat: roles of descending noradrenergic and serotonergic systems. J Pharmacol Sci.

[120] De Reuck J, Decoo D, Vanderdonckt P, Dallenga A, Ceusters W, Kalala JP (1994). A double-blind study of neurotropin in patients with acute ischemic stroke. Acta Neurol Scand.

[121] Malone K, Amu S, Moore AC, Waeber C (2019). The immune system and stroke: from current targets to future therapy. Immunol Cell Biol.

[122] Vogelgesang A, Becker KJ, Dressel A (2014). Immunological consequences of ischemic stroke. Acta Neurol Scand.

[123] Anrather J, Iadecola C (2016). Inflammation and stroke: an overview. Neurotherapeutics.

[124] Selvaraj UM, Poinsatte K, Torres V, Ortega SB, Stowe AM (2016). Heterogeneity of b cell functions in stroke-related risk, prevention, injury, and repair. Neurotherapeutics.

[125] Iadecola C, Anrather J (2011). The immunology of stroke: from mechanisms to translation. Nat Med.

[126] Brambilla R, Couch Y, Lambertsen KL (2013). The effect of stroke on immune function. Mol Cell Neurosci.

[127] Muscari A, Puddu GM, Santoro N, Serafini C, Cenni A, Rossi V (2011). The atorvastatin during ischemic stroke study: a pilot randomized controlled trial. Clin Neuropharmacol.

[128] Westendorp WF, Vermeij JD, Zock E, Hooijenga IJ, Kruyt ND, Bosboom HJ (2015). The preventive antibiotics in stroke study (pass): a pragmatic randomised open-label masked endpoint clinical trial. Lancet.

[129] Piot C, Croisille P, Staat P, Thibault H, Rioufol G, Mewton N (2008). Effect of cyclosporine on reperfusion injury in acute myocardial infarction. N Engl J Med.

[130] Borlongan CV, Yu G, Matsukawa N, Xu L, Hess DC, Sanberg PR (2005). Acute functional effects of cyclosporine-a and methylprednisolone treatment in adult rats exposed to transient ischemic stroke. Life Sci.

[131] Schinzel AC, Takeuchi O, Huang Z, Fisher JK, Zhou Z, Rubens J (2005). Cyclophilin d is a component of mitochondrial permeability transition and mediates neuronal cell death after focal cerebral ischemia. Proc Natl Acad Sci USA.

[132] Nighoghossian N, Berthezene Y, Mechtouff L, Derex L, Cho TH, Ritzenthaler T (2015). Cyclosporine in acute ischemic stroke. Neurology.

[133] Bowes MP, Rothlein R, Fagan SC, Zivin JA (1995). Monoclonal antibodies preventing leukocyte activation reduce experimental neurologic injury and enhance efficacy of thrombolytic therapy. Neurology.

[134] Clark WM, Madden KP, Rothlein R, Zivin JA (1991). Reduction of central nervous system ischemic injury by monoclonal antibody to intercellular adhesion molecule. J Neurosurg.

[135] Zhang RL, Chopp M, Li Y, Zaloga C, Jiang N, Jones ML (1994). Anti-icam-1 antibody reduces ischemic cell damage after transient middle cerebral artery occlusion in the rat. Neurology.

[136] Enlimomab Acute Stroke Trial I (2001). Use of anti-icam-1 therapy in ischemic stroke: results of the enlimomab acute stroke trial. Neurology.

[137] Zhu Z, Fu Y, Tian D, Sun N, Han W, Chang G (2015). Combination of the immune modulator fingolimod with alteplase in acute ischemic stroke: a pilot trial. Circulation.

[138] Massberg S, von Andrian UH (2006). Fingolimod and sphingosine-1-phosphate–modifiers of lymphocyte migration. N Engl J Med.

[139] Lampl Y, Boaz M, Gilad R, Lorberboym M, Dabby R, Rapoport A (2007). Minocycline treatment in acute stroke: an open-label, evaluator-blinded study. Neurology.

[140] Kohler E, Prentice DA, Bates TR, Hankey GJ, Claxton A, van Heerden J (2013). Intravenous minocycline in acute stroke: a randomized, controlled pilot study and meta-analysis. Stroke.

[141] Harms H, Prass K, Meisel C, Klehmet J, Rogge W, Drenckhahn C (2008). Preventive antibacterial therapy in acute ischemic stroke: a randomized controlled trial. PLoS ONE.

[142] Becker K, Kindrick D, Relton J, Harlan J, Winn R (2001). Antibody to the alpha4 integrin decreases infarct size in transient focal cerebral ischemia in rats. Stroke.

[143] Langhauser F, Kraft P, Gob E, Leinweber J, Schuhmann MK, Lorenz K (2014). Blocking of alpha4 integrin does not protect from acute ischemic stroke in mice. Stroke.

[144] Liesz A, Zhou W, Mracsko E, Karcher S, Bauer H, Schwarting S (2011). Inhibition of lymphocyte trafficking shields the brain against deleterious neuroinflammation after stroke. Brain.

[145] Llovera G, Hofmann K, Roth S, Salas-Perdomo A, Ferrer-Ferrer M, Perego C (2015). Results of a preclinical randomized controlled multicenter trial (prct): anti-cd49d treatment for acute brain ischemia. Sci Transl Med..

[146] Relton JK, Sloan KE, Frew EM, Whalley ET, Adams SP, Lobb RR (2001). Inhibition of alpha4 integrin protects against transient focal cerebral ischemia in normotensive and hypertensive rats. Stroke.

[147] Elkins J, Veltkamp R, Montaner J, Johnston SC, Singhal AB, Becker K (2017). Safety and efficacy of natalizumab in patients with acute ischaemic stroke (action): a randomised, placebo-controlled, double-blind phase 2 trial. Lancet Neurol.

[148] Montaner J, Chacon P, Krupinski J, Rubio F, Millan M, Molina CA (2008). Simvastatin in the acute phase of ischemic stroke: a safety and efficacy pilot trial. Eur J Neurol.

[149] Montaner J, Bustamante A, Garcia-Matas S, Martinez-Zabaleta M, Jimenez C, de la Torre J (2016). Combination of thrombolysis and statins in acute stroke is safe: results of the stars randomized trial (stroke treatment with acute reperfusion and simvastatin). Stroke.

[150] Jiang N, Chopp M, Chahwala S (1998). Neutrophil inhibitory factor treatment of focal cerebral ischemia in the rat. Brain Res.

[151] Krams M, Lees KR, Hacke W, Grieve AP, Orgogozo JM, Ford GA (2003). Acute stroke therapy by inhibition of neutrophils (astin): an adaptive dose-response study of uk-279,276 in acute ischemic stroke. Stroke.

[152] den Hertog HM, van der Worp HB, van Gemert HM, Algra A, Kappelle LJ, van Gijn J (2009). The paracetamol (acetaminophen) in stroke (pais) trial: a multicentre, randomised, placebo-controlled, phase iii trial. Lancet Neurol.

[153] Koennecke HC, Leistner S (2001). Prophylactic antipyretic treatment with acetaminophen in acute ischemic stroke: a pilot study. Neurology.

[154] Diener HC, Schneider D, Lampl Y, Bornstein NM, Kozak A, Rosenberg G (2008). Dp-b99, a membrane-activated metal ion chelator, as neuroprotective therapy in ischemic stroke. Stroke.

[155] Teal P, Davis S, Hacke W, Kaste M, Lyden PD, Modified Randomized Exposure Controlled Trial Study I (2009). A randomized, double-blind, placebo-controlled trial to evaluate the efficacy, safety, tolerability, and pharmacokinetic/pharmacodynamic effects of a targeted exposure of intravenous repinotan in patients with acute ischemic stroke: modified randomized exposure controlled trial (mrect). Stroke.

[156] Chen C, Li M, Lin L, Chen S, Chen Y, Hong L (2021). Clinical effects and safety of edaravone in treatment of acute ischaemic stroke: a meta-analysis of randomized controlled trials. J Clin Pharm Ther.

[157] Hu R, Guo Y, Lin Y, Tang Y, Tang Q, Wang X (2021). Safety and efficacy of edaravone combined with alteplase for patients with acute ischemic stroke: a systematic review and meta-analysis. Pharmazie.

[158] Yang B, Shi J, Chen X, Ma B, Sun H (2014). Efficacy and safety of therapies for acute ischemic stroke in china: a network meta-analysis of 13289 patients from 145 randomized controlled trials. PLoS ONE.

[159] Yang J, Cui X, Li J, Zhang C, Zhang J, Liu M (2015). Edaravone for acute stroke: meta-analyses of data from randomized controlled trials. Dev Neurorehabil.

[160] Bailly C, Hecquet PE, Kouach M, Thuru X, Goossens JF (2020). Chemical reactivity and uses of 1-phenyl-3-methyl-5-pyrazolone (pmp), also known as edaravone. Bioorg Med Chem.

[161] Ren Y, Wei B, Song X, An N, Zhou Y, Jin X (2015). Edaravone’s free radical scavenging mechanisms of neuroprotection against cerebral ischemia: review of the literature. Int J Neurosci.

[162] Yoshida H, Yanai H, Namiki Y, Fukatsu-Sasaki K, Furutani N, Tada N (2006). Neuroprotective effects of edaravone: a novel free radical scavenger in cerebrovascular injury. CNS Drug Rev.

[163] Feng S, Yang Q, Liu M, Li W, Yuan W, Zhang S (2011). Edaravone for acute ischaemic stroke. Cochrane Database Syst Rev.

[164] Kaste M, Murayama S, Ford GA, Dippel DW, Walters MR, Tatlisumak T (2013). Safety, tolerability and pharmacokinetics of mci-186 in patients with acute ischemic stroke: new formulation and dosing regimen. Cerebrovasc Dis.

[165] Kikuchi K, Takeshige N, Miura N, Morimoto Y, Ito T, Tancharoen S (2012). Beyond free radical scavenging: beneficial effects of edaravone (radicut) in various diseases (review). Exp Ther Med.

[166] Levard D, Buendia I, Lanquetin A, Glavan M, Vivien D, Rubio M (2020). Filling the gaps on stroke research: focus on inflammation and immunity. Brain Behav Immun.

[167] Ahmed N, Wahlgren NG (2003). Effects of blood pressure lowering in the acute phase of total anterior circulation infarcts and other stroke subtypes. Cerebrovasc Dis.

[168] Gasecki D, Kwarciany M, Kowalczyk K, Narkiewicz K, Karaszewski B (2020). Blood pressure management in acute ischemic stroke. Curr Hypertens Rep.

[169] Mohr JP, Orgogozo JM, Harrison MJG, Hennerici M, Wahlgren NG, Gelmers JH (1994). Metaanalysis of oral nimodipine trials in acute ischemic stroke. Cerebrovasc Dis.

[170] Yeo SH, Lim ZI, Mao J, Yau WP (2017). Effects of central nervous system drugs on recovery after stroke: a systematic review and meta-analysis of randomized controlled trials. Clin Drug Investig.

[171] Muir KW (2002). Heterogeneity of stroke pathophysiology and neuroprotective clinical trial design. Stroke.

[172] Catanese L, Tarsia J, Fisher M (2017). Acute ischemic stroke therapy overview. Circ Res.

[173] Cho TH, Nighoghossian N, Mikkelsen IK, Derex L, Hermier M, Pedraza S (2015). Reperfusion within 6 hours outperforms recanalization in predicting penumbra salvage, lesion growth, final infarct, and clinical outcome. Stroke.

[174] Eilaghi A, Brooks J, d'Esterre C, Zhang L, Swartz RH, Lee TY (2013). Reperfusion is a stronger predictor of good clinical outcome than recanalization in ischemic stroke. Radiology.

[175] Makris N, Chamard L, Mikkelsen IK, Hermier M, Derex L, Pedraza S (2019). Acute reperfusion without recanalization: serial assessment of collaterals within 6 h of using perfusion-weighted magnetic resonance imaging. J Cereb Blood Flow Metab.

[176] Lapergue B, Blanc R, Gory B, Labreuche J, Duhamel A, Marnat G (2017). Effect of endovascular contact aspiration vs stent retriever on revascularization in patients with acute ischemic stroke and large vessel occlusion: the aster randomized clinical trial. JAMA.

[177] Powers WJ, Rabinstein AA, Ackerson T, Adeoye OM, Bambakidis NC, Becker K (2019). Guidelines for the early management of patients with acute ischemic stroke: 2019 update to the 2018 guidelines for the early management of acute ischemic stroke: a guideline for healthcare professionals from the american heart association/american stroke association. Stroke.

[178] Furlan A, Higashida R, Wechsler L, Gent M, Rowley H, Kase C (1999). Intra-arterial prourokinase for acute ischemic stroke. The proact ii study: a randomized controlled trial. Prolyse in acute cerebral thromboembolism. JAMA.

[179] Broderick JP, Palesch YY, Demchuk AM, Yeatts SD, Khatri P, Hill MD (2013). Endovascular therapy after intravenous t-pa versus t-pa alone for stroke. N Engl J Med.

[180] Ciccone A, Valvassori L, Investigators SE (2013). Endovascular treatment for acute ischemic stroke. N Engl J Med.

[181] Kidwell CS, Jahan R, Gornbein J, Alger JR, Nenov V, Ajani Z (2013). A trial of imaging selection and endovascular treatment for ischemic stroke. N Engl J Med.

[182] Albers GW, Marks MP, Kemp S, Christensen S, Tsai JP, Ortega-Gutierrez S (2018). Thrombectomy for stroke at 6 to 16 hours with selection by perfusion imaging. N Engl J Med.

[183] Nogueira RG, Jadhav AP, Haussen DC, Bonafe A, Budzik RF, Bhuva P (2018). Thrombectomy 6 to 24 hours after stroke with a mismatch between deficit and infarct. N Engl J Med.

[184] Furlan AJ (2012). Challenges in acute ischemic stroke clinical trials. Curr Cardiol Rep.

[185] Kikuchi K, Tanaka E, Murai Y, Tancharoen S (2014). Clinical trials in acute ischemic stroke. CNS Drugs.

[186] Antonic A, Dottori M, Macleod MR, Donnan GA, Howells DW (2018). Nxy-059, a failed stroke neuroprotectant, offers no protection to stem cell-derived human neurons. J Stroke Cerebrovasc Dis.

[187] Kuroda S, Tsuchidate R, Smith ML, Maples KR, Siesjo BK (1999). Neuroprotective effects of a novel nitrone, nxy-059, after transient focal cerebral ischemia in the rat. J Cereb Blood Flow Metab.

[188] Wu Q, Yan R, Sun J (2020). Probing the drug delivery strategies in ischemic stroke therapy. Drug Deliv.

[189] O'Collins VE, Macleod MR, Donnan GA, Horky LL, van der Worp BH, Howells DW (2006). 1,026 experimental treatments in acute stroke. Ann Neurol.

[190] Baron JC (2018). Protecting the ischaemic penumbra as an adjunct to thrombectomy for acute stroke. Nat Rev Neurol.

[191] Auer RN (1995). Combination therapy with u74006f (tirilazad mesylate), mk-801, insulin and diazepam in transient forebrain ischaemia. Neurol Res.

[192] Lyden P, Lonzo L, Nunez S (1995). Combination chemotherapy extends the therapeutic window to 60 minutes after stroke. J Neurotrauma.

[193] Schabitz WR, Li F, Irie K, Sandage BW, Locke KW, Fisher M (1999). Synergistic effects of a combination of low-dose basic fibroblast growth factor and citicoline after temporary experimental focal ischemia. Stroke.

[194] Uematsu D, Araki N, Greenberg JH, Sladky J, Reivich M (1991). Combined therapy with mk-801 and nimodipine for protection of ischemic brain damage. Neurology.

[195] Bogousslavsky J, Regli F, Zumstein V, Kobberling W (1990). Double-blind study of nimodipine in non-severe stroke. Eur Neurol.

[196] Randomised, double-blind, placebo-controlled trial of nimodipine in acute stroke. Trust study group. Lancet. 1990;336:1205–1209. 1978069

[197] Clinical trial of nimodipine in acute ischemic stroke (1992). The American nimodipine study group. Stroke.

[198] Martinez-Vila E, Guillen F, Villanueva JA, Matias-Guiu J, Bigorra J, Gil P (1990). Placebo-controlled trial of nimodipine in the treatment of acute ischemic cerebral infarction. Stroke.

[199] Fogelholm R, Erila T, Palomaki H, Murros K, Kaste M (2000). Effect of nimodipine on final infarct volume after acute ischemic stroke. Cerebrovasc Dis.

[200] Horn J, de Haan RJ, Vermeulen M, Limburg M (2001). Very early nimodipine use in stroke (venus): a randomized, double-blind, placebo-controlled trial. Stroke.

